# Association of predicted pathogenic mutations in mitochondrial *ND* genes with distant metastasis in NSCLC and colon cancer

**DOI:** 10.1038/s41598-017-15592-2

**Published:** 2017-11-14

**Authors:** Nobuko Koshikawa, Miho Akimoto, Jun-Ichi Hayashi, Hiroki Nagase, Keizo Takenaga

**Affiliations:** 1 Laboratory of Cancer Genetics, Chiba Cancer Center Research Institute, 666-2 Nitona, Chuoh-ku, Chiba, 260-8717 Japan; 2 Department of Life Science, Shimane University Faculty of Medicine, 89-1 Enya, Izumo, Shimane 693-8501 Japan; 3 University of Tsukuba, 1-1-1 Tennodai, Tsukuba, Ibaraki, 305-8572 Japan; 4 Present Address: Department of Biochemistry, Teikyo University School of Medicine, 2–11-1 Kaga, Itabashi-ku, Tokyo 173–8605 Japan

## Abstract

Cancer cells have more mutations in their mitochondrial DNA (mtDNA) than do normal cells, and pathogenic mutations in the genes encoding mitochondrial NADH dehydrogenase (ND) subunits have been found to enhance the invasive and metastatic ability of various tumour cells in animal experiments. However, it is unknown whether single-nucleotide variants (SNVs) of the *ND* genes that decrease complex I activity are involved in distant metastasis in human clinical samples. Here, we demonstrated the enhancement of the distant metastasis of Lewis lung carcinoma cells by the *ND6* 13885insC mutation, which is accompanied by the overexpression of metastasis-related genes, metabolic reprogramming, the enhancement of tumour angiogenesis and the acquisition of resistance to stress-induced cell death. We then sequenced *ND* genes in primary tumour lesions with or without distant metastases as well as metastatic tumour lesions from 115 patients with non-small cell lung cancer (NSCLC) and colon cancer, and we subsequently selected 14 SNVs with the potential to decrease complex I activity. Intriguingly, a significant correlation was observed (*P* < 0.05 by Chi-square test) between the incidence of the selected mutations and distant metastasis. Thus, these results strongly suggest that pathogenic *ND* gene mutations participate in enhancing distant metastasis in human cancers.

## Introduction

Mitochondria are the key regulators of the oxidative phosphorylation system, which consists of five complexes (I-V). Mitochondria have their own genome, mitochondrial DNA (mtDNA), which contains 37 genes, including seven subunits of complex I (NADH dehydrogenase (ND)1, ND2, ND3, ND4L, ND4, ND5 and ND6), one subunit of complex III (cytochrome b (CYTB)) and three subunits of complex IV (Cyt c oxidase (CO) I, II and III).

Mitochondrial complex I is the large membrane protein complex of the respiratory chain^1^, whose central subunits are well conserved from bacteria to humans. It functions as a proton-pumping NADH:ubiquinone oxidoreductase and couples the electron transfer from NADH (reduced nicotine adenine dinucleotide) to ubiquinone, thereby translocating four protons from the mitochondrial matrix to the intermembrane space. Analysis of the structure of complex I of *Yarrowia lipolytica* and *Bos Taurus* heart has revealed that the complex consists of a hydrophilic peripheral arm and a hydrophobic membrane arm, which form an L-shaped structure^1,2^. The peripheral arm extends into the matrix and is oriented perpendicular to the membrane arm, which consists of the distal P_D_ module (ND5 and ND4) and the proximal P_P_ module (ND2, ND4L, ND6, ND3 and ND1). The peripheral arm comprises the N module and the Q module: the Q module docks the N module onto the membrane arm, forming an electron transfer path. ND1, at the proximal end of the membrane arm, provides the docking site for the Q module. Complex I is a crucial component in the respiratory chain, the maintenance of the NAD^+^/NADH balance and reactive oxygen species (ROS) levels, the generation of mitochondrial membrane potential and ATP production, and therefore, its dysfunction is often the cause of mitochondrial disorders and diseases^3–5^.

Somatic mutations in mtDNA have been shown to accumulate in cancer cells and have been proposed to contribute to carcinogenesis and the malignant progression of cancers with a variety of tissue origins^4^. In particular, mutations in the *ND* genes affect malignant behaviour such as the invasion and metastasis of cancer cells. We have previously demonstrated that certain ROS-generating mtDNA mutations in the *ND6* gene, G13997A and 13885insC, enhance metastasis in low-metastatic Lewis lung carcinoma cells and fibrosarcoma cells, respectively^6,7^, providing the first report of the involvement of pathogenic *ND* gene mutations in metastasis. Thereafter, a few groups reported similar effects of pathogenic *ND* gene mutations on invasion and metastasis; for example, the *ND5* G13289A mutation increases ROS production, cell proliferation and invasion in human lung cancer cells^8^. The *ND3* G10398A mutation enhances the invasion and metastasis of human breast cancer cells in a xenograft model, and *ND6* missense and nonsense mutations have the same effect *in vitro* ^9,10^. The frequency of *ND6* gene mutations correlates with lymph node metastasis in patients with lung adenocarcinoma^10^. In contrast, although *ND* mutations are not involved, increasing complex I activity by manipulating the NAD^+^/NADH balance has been found to decrease breast cancer metastasis in a xenograft model^11^. Thus, aberrations in complex I activity are likely to be associated with invasion and metastasis. However, it is uncertain why reduction of complex I activity is mostly reported to be associated with invasion and metastasis. Furthermore, it remains unclear whether *ND* gene mutations, which decrease complex I activity, are indeed associated with distant metastasis in human cancers. One of the problems in investigating this association is that many different somatic mutations occur randomly in the *ND* genes in cancer cells, and even missense mutations do not all decrease complex I activity. The only way to demonstrate the reliable and tight correlation between a mutation and a decrease in complex I activity is to establish a cybrid line with a somatic mutation and to compare the activity between the cybrid and the control cybrid with wild-type mtDNA and, importantly, the same nuclear background. However, it is enormously laborious to check the pathogenicity of all mutations found in cancer cells. Thus, in this study, we sought to predict the pathogenicity of *ND* gene mutations found in clinical cancer specimens and investigate the relationship between their incidence and distant metastasis. We focused on nonsynonymous single-nucleotide variants (SNVs) and single-nucleotide polymorphisms (SNPs) (SNVs observed in at least 1% of the population were defined as SNPs in this study) in the *ND* genes of non-small cell lung cancer (NSCLC) and colon cancer and selected candidate SNVs and SNPs with a high probability of reducing complex I activity, on the basis of the Grantham value; the evolutionary conservation of the original amino acid residue; the effect of the altered amino acid residue on protein structure; reported disease associations; and the predicted pathogenicity score. The results showed that the incidence of the selected nonsynonymous SNVs and SNPs predicted to decrease complex I activity was significantly associated with distant metastasis.

## Results

### *ND6* 13885insC mutation enhances metastasis of low-metastatic cells

**Figure 1:**
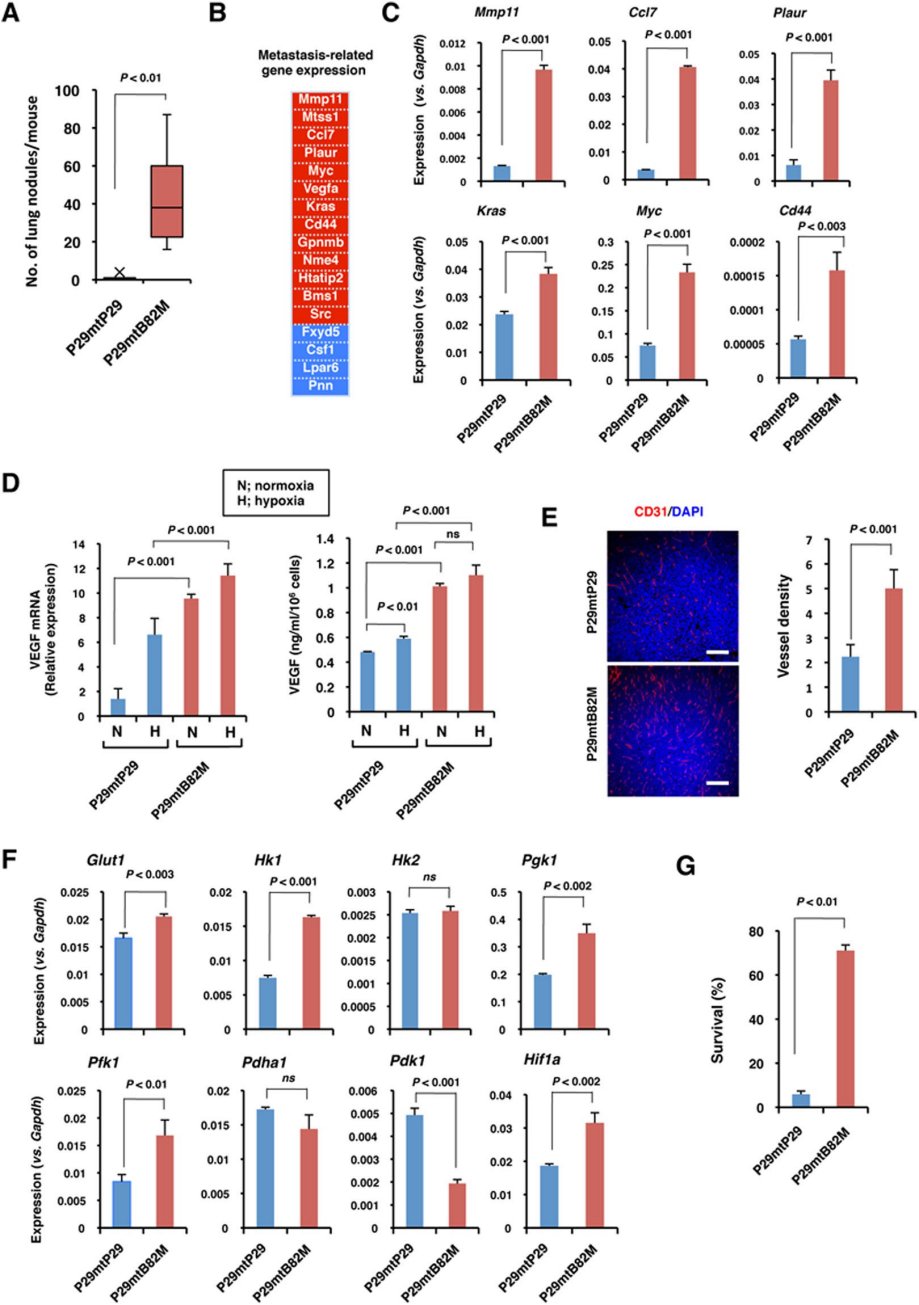
Metastasis-related characteristics of P29mtB82M cybrids harbouring an *ND6* 13885insC mutation. (**A**) Spontaneous lung metastatic ability of P29mtP29 (n = 6 mice) and P29mtB82M cells (n = 7 mice). (**B**) Profiling of metastasis-related genes examined with a Mouse Metastasis PCR Array. Upregulated (red, >2-fold expression) and downregulated (blue, >2-fold expression) genes in P29mtB82M cells are shown. (**C**) qRT-PCR analyses of some of the genes differentially expressed in P29mtB82M cells. The relative level of gene expression in P29mtP29 and P29mtB82M cells was normalized against that of mouse *Gapdh*. (**D**) qRT-PCR analysis of the expression of *vegfa* mRNA (left) and ELISA analysis of VEGF-A protein (right) under normoxic and hypoxic (1% O_2_) conditions. (**E**) Tumour angiogenesis in P29mtP29 and P29mtB82M tumours as assessed by CD31 staining (left) and vessel density (right). Scale bars; 200 μm. (**F)** qRT-PCR analysis of the expression of glycolytic enzymes, *Pdha1*, *Pdk1* and *Hif1a*. (**G**) Survival of P29mtP29 and P29mtB82M cells in severe hypoxia. The cells were incubated in < 0.1% O_2_ for 2 days.

We have previously shown that P29mtB82M cells harbouring a 13885insC mutation in the *ND6* gene show lower complex I activity, produce a larger amount of ROS, and exhibit higher lung-colonizing ability than do P29mtP29 cells with wild-type mtDNA^6^. Here, we further characterized the phenotypes of P29mtB82M cells, including their spontaneous metastatic potential. P29mtB82M cells formed a larger number of spontaneous metastatic foci than did P29mtP29 cells (Fig. 1A). Furthermore, PCR array analysis of the expression of metastasis-related genes revealed the upregulation of matrix metalloproteinase 11 (*Mmp11*), urokinase-type plasminogen activator receptor (*Plaur*), C-C chemokine ligand 7 (monocyte chemotactic protein 3) (*Ccl7*), *c-Myc*, *K-ras*, *Cd44* and vascular endothelial growth factor-A (*Vegfa*), which were confirmed by qRT-PCR (Fig. 1B,C). VEGF-A expression was further enhanced under hypoxic conditions at both the mRNA and protein levels in P29mtP29 and P29mtB82M cells, and the latter showed significantly higher VEGF-A expression than the former (Fig. 1D). Correspondingly, P29mtB82M tumours had higher vessel density than did P29mtP29 tumours (Fig. 1E). Impeding mitochondrial function causes a shift in the energy metabolism towards enhanced aerobic glycolysis^12^. Accordingly, the expression levels of the genes encoding glucose transporter 1 (*Glut1*), hexokinase 1 (*Hk1*), phosphoglycerate kinase 1 (*Pgk1*) and phosphofructokinase 1 (*Pfk1*), but not *Hk2* or pyruvate dehydrogenase α1 (*Pdha1*), were upregulated. Interestingly, we found that the expression of pyruvate dehydrogenase kinase 1 (*Pdk1*), which inactivates the TCA cycle enzyme PDH, which converts pyruvate to acetyl-CoA^13^, was suppressed in P29mtB82M cells (Fig. 1F). The expression of hypoxia-inducible factor-1α (*Hif1a*), which is involved in regulating the expression of *Vegf*, *Glut1*, *Hk1*, *Pgk1* and *Pfk1*, was increased in P29mtB82M cells. HIF-1 stimulates cell survival under hypoxic conditions^14^. Indeed, P29mtB82M cells were more resistant to severe hypoxia (Fig. 1G). Thus, introducing the *ND6* 13885insC mutation appeared to enhance the distant metastasis of low-metastatic Lewis lung carcinoma cells by stimulating the expression of metastasis-related genes, metabolic reprogramming and tumour angiogenesis and by conferring resistance to stress-induced cell death.

### SNVs and SNPs in the ND genes in tissues of NSCLC and colon cancer

Because mutations occur randomly in mtDNA, it is natural to consider that the types of mtDNA mutation vary enormously for each cancer cell, and therefore, there may be no common mutation associated with metastasis. However, the above results and previous reports^6,7,9,11^ demonstrating the importance of *ND* gene mutations that modulate complex I activity for distant metastasis prompted us to seek clues as to whether pathogenic missense and nonsense mutations in the *ND* genes are involved in the distant metastasis of human cancers. To this end, we sequenced the *ND1*, *ND2*, *ND3*, *ND4L*, *ND4*, *ND5* and *ND6* genes in the tissues of 45 primary tumours (1 case with distant metastasis and 44 distant metastasis-free cases) and 37 brain metastases of NSCLCs and in the tissues of 22 primary tumours (7 cases with distant metastasis and 15 distant metastasis-free cases) and 11 distant metastases of colon cancers, including two matched primary and metastasis samples (Supplementary Tables S1 and S3). Nonsynonymous and synonymous SNVs and SNPs are shown in Supplementary Tables S1–S4. A total of 51 somatic mutations (22 nonsynonymous and 29 synonymous) were found in the specimens (Table 1, Supplementary Tables S2 and S4). Interestingly, a higher somatic mutation frequency per unit length was observed in the *ND6* gene than in other *ND* genes and in the *COII*, *ATP6* and *CYTB* genes, which were also sequenced for comparison (Table 1, Supplementary Tables S1 and S3). Some of the mutations were detected as overlapping peaks at the same position, thus indicating heteroplasmy (or a mixture of cancer cells harbouring the mutation and stromal cells), whereas the others were detected as a single peak, thus indicating homoplasmy, on the sequence electropherogram (Supplementary Figure S1, Supplementary Tables S2 and S4). The ratio of homoplasmy/heteroplasmy was 5/9 (35.7%) in distant metastastic lesions and 87/8 (91.6%) in primary lesions, indicating that homoplasmic states are less prevalent in metastatic lesions.

**Table 1:** Frequency of somatic mutations and the occurrence of SNVs and SNPs that meet the criteria for pathogenicity in the mtDNA genes.

mtDNA gene		No. of somatic mutations (Average No. per 100 bp)			No. of SNVs and SNPs that meet the criteria for pathogenicity (Average No. per 100 bp)
Name	Size (bp)	Nonsynonymous	Synonymous	Total	Total
*ND1*	956	4 (0.418)	5 (0.628)	9 (0.941)	6 (0.628)
*ND2*	1,042	2 (0.192)	2 (0.192)	4 (0.384)	0(0)
*ND3*	346	2 (0.578)	0(0)	2 (0.578)	1 (0.289)
*ND4L*	297	0(0)	1 (0.337)	1 (0.337)	0(0)
*ND4*	1,378	3 (0.218)	6 (0.435)	9 (0.653)	1 (0.073)
*ND5*	1,812	7 (0.386)	9 (0.497)	16 (0.883)	5 (0.276)
*ND6*	525	4 (0.762)	6 (1.143)	10 (1.905)	1 (0.19)
Total	6356	22 (0.346)	29 (0.456)	51 (0.802)	14 (0.22)
*COII*	684	2 (0.293)	5 (0.731)	7 (1.023)	0(0)
*ATP6*	681	3 (0.441)	4 (0.587)	7 (1.028)	0(0)
*CYTB*	1,141	6 (0.526)	0(0)	6 (0.526)	1 (0.088)
Total	2,506	11 (0.439)	9 (0.359)	20 (0.798)	1 (0.04)

**Figure 2:**
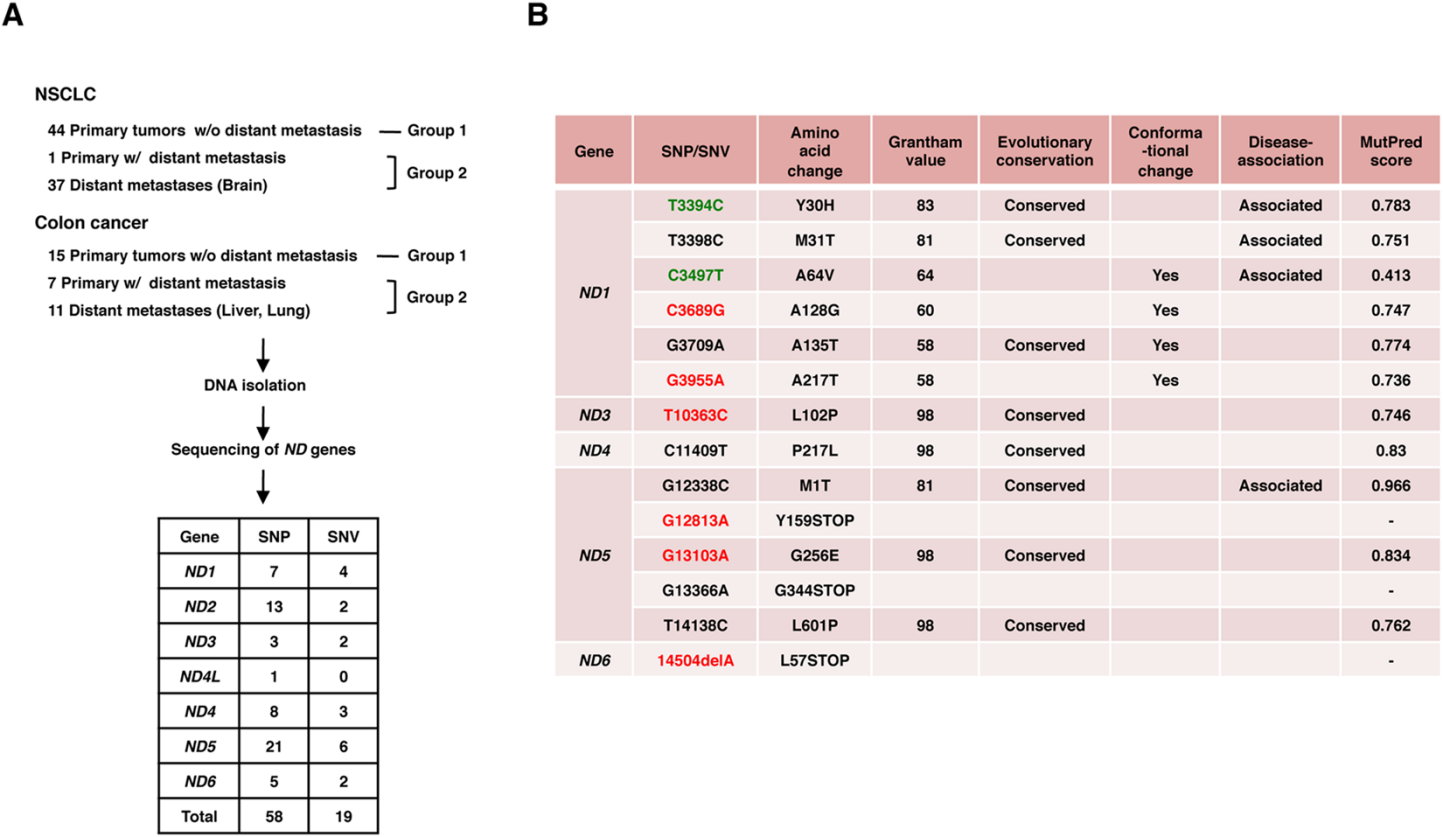
Analysis of mtDNA SNVs in clinical specimens. (**A**) The number and grouping of NSCLC and colon cancer cases subjected to analysis and the number of SNPs and SNVs detected. (**B**) Properties of each of the selected SNPs and SNVs. SNPs and novel mutations are indicated in green and red, respectively.

**Figure 3:**
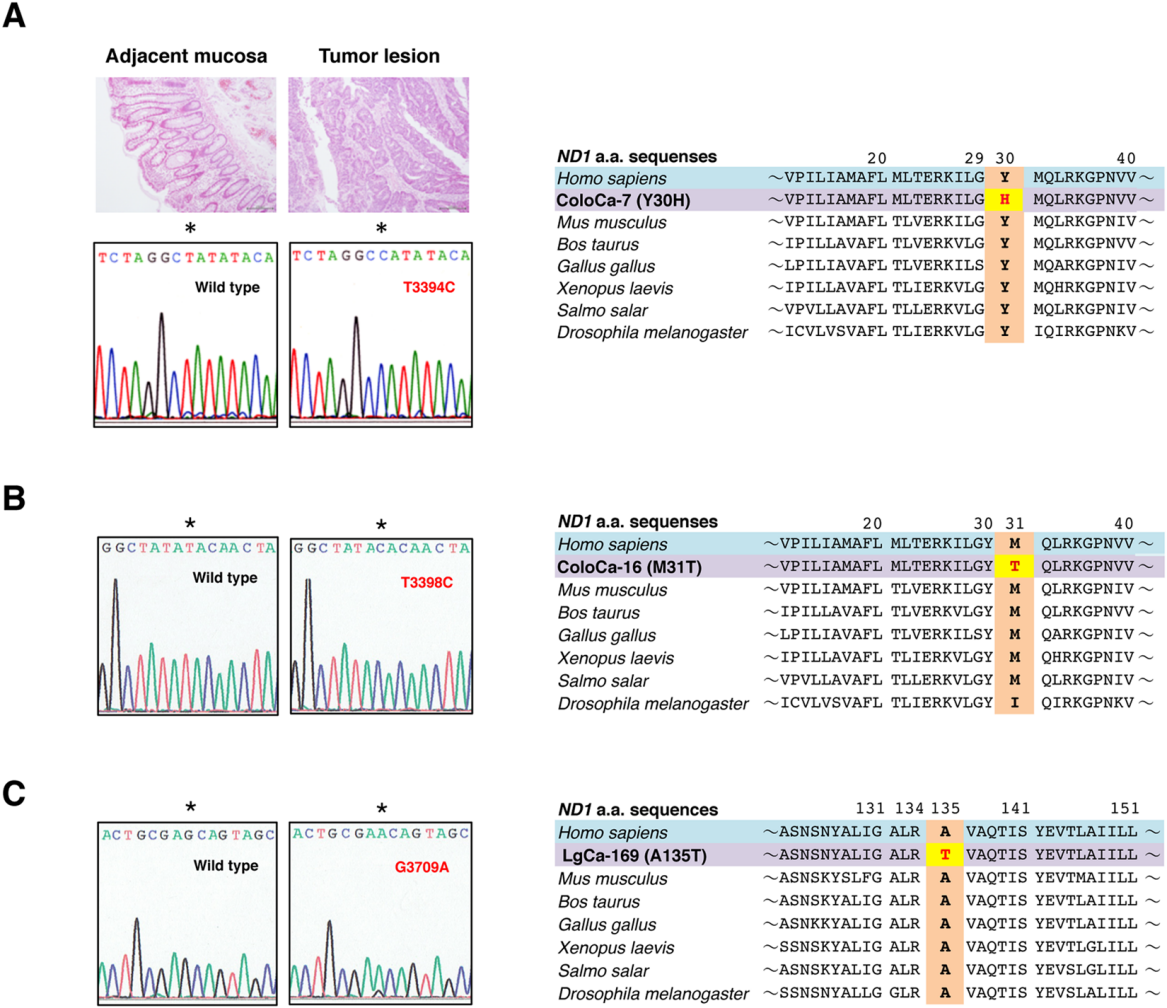
Mutations in the *ND1* gene that alter evolutionarily conserved amino acid residues. (**A**) Images of haematoxylin and eosin (HE) staining of tumour lesion and adjacent normal tissues of colon cancer (ColoCa-7) are shown. Electropherograms depict the T3394C (Y30H) SNV. The SNV is present in the tumour lesion but not in the adjacent mucosal tissue. The evolutionary conservation of the tyrosine residue is shown. (**B**) Electropherograms showing the T3398C (M31T) SNV. The evolutionary conservation of the histidine residue is shown. (**C**) Electropherograms showing the G3709A (A135T) SNV. The evolutionary conservation of the alanine residue is shown.

**Figure 4:**
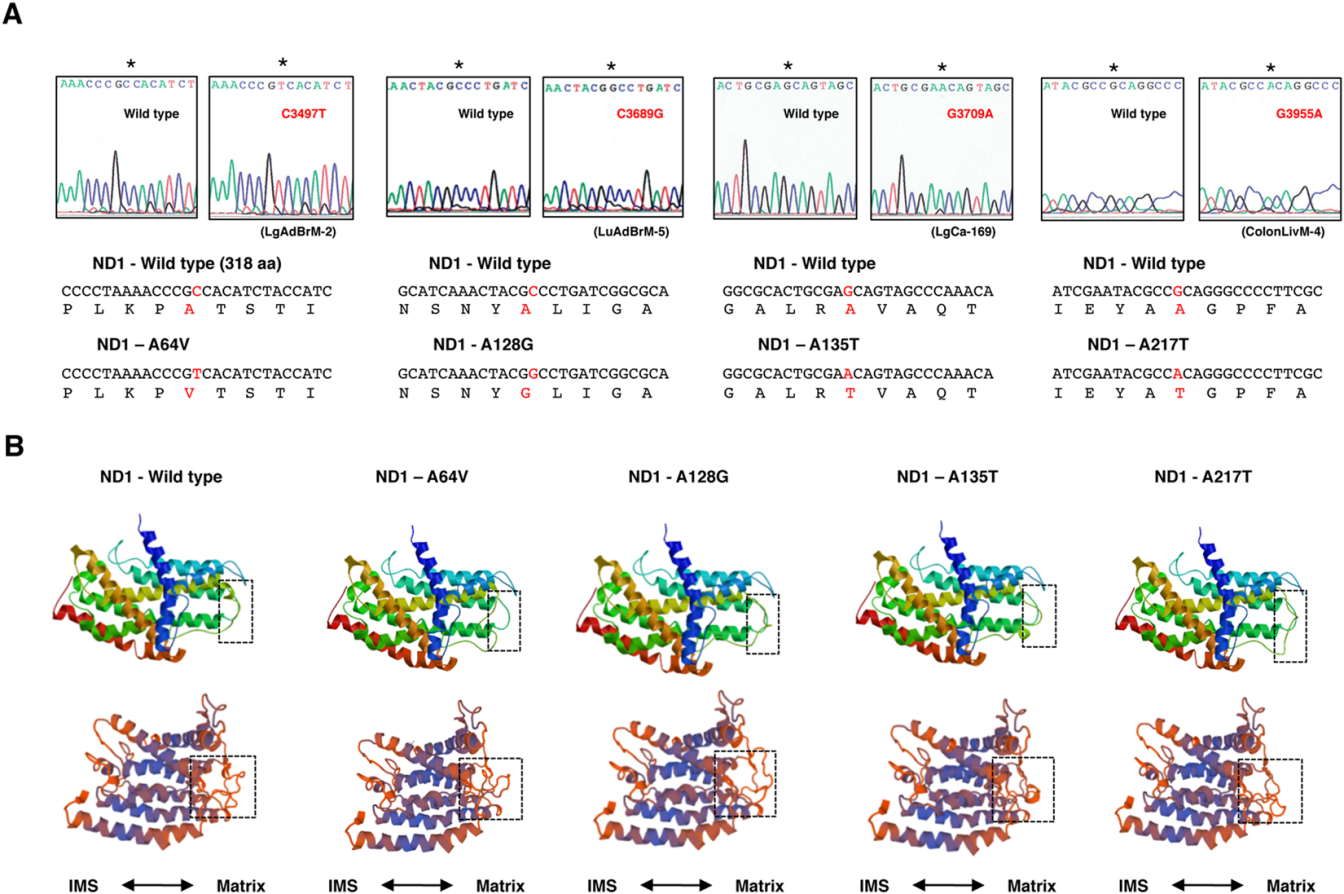
Mutations in the *ND1* gene that change the conformation of the ND1 protein, as predicted by SWISS-MODEL. (**A**) Electropherograms showing the SNVs and the amino acid changes caused by the SNVs. (**B**) Upper: Rectangles with a dashed border indicate the areas containing the conformational change caused by the mutation. Bottom: The image from a different angle. IMS: intermembrane space.

**Figure 5:**
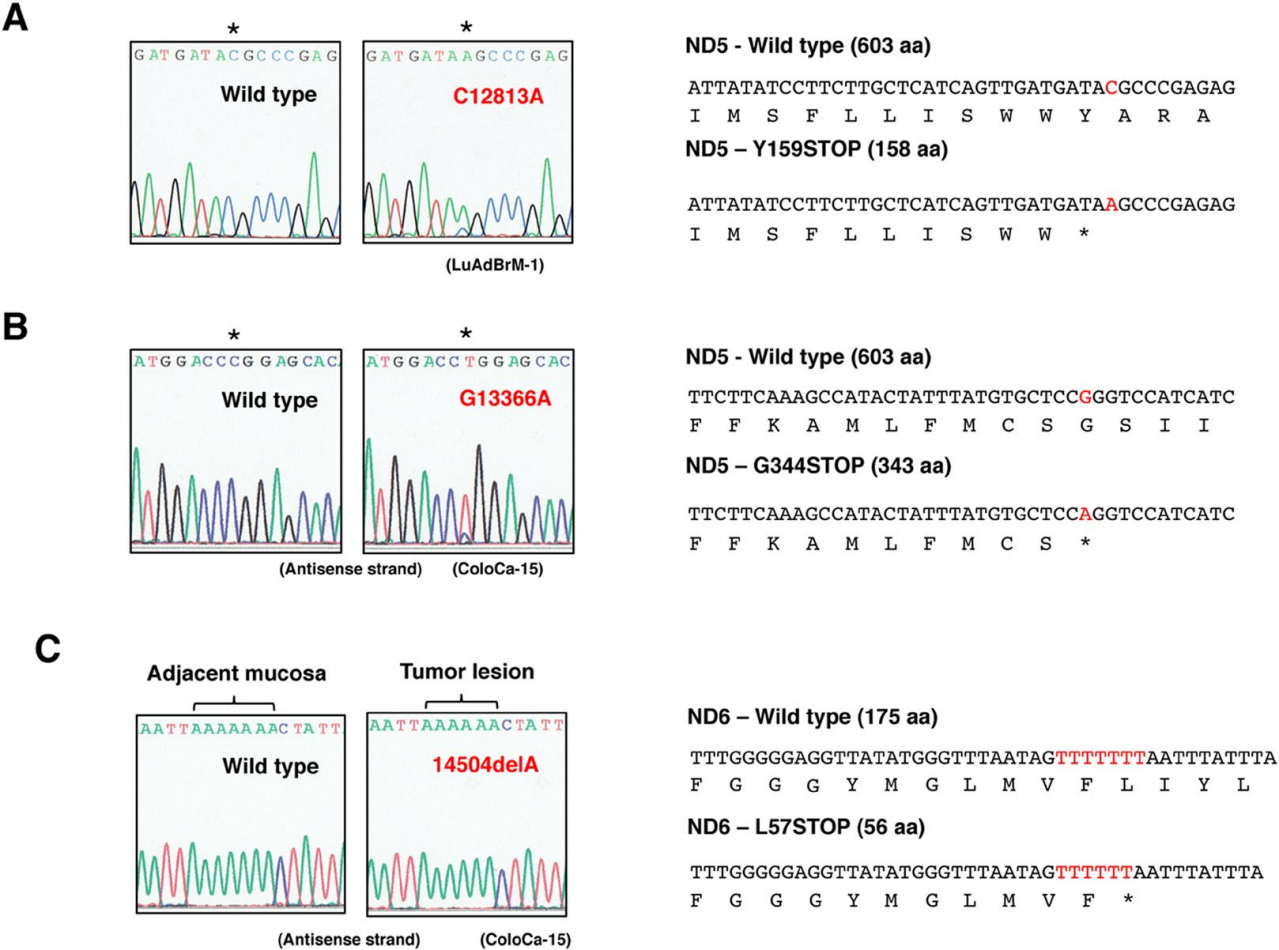
Nonsense mutations detected in the *ND5* and *ND6* genes. Electrophoregrams and the changes in the amino acid sequence are shown. (**A**) C12813A mutation in the *ND5* gene. (**B**) G13366A mutation in the *ND5* gene. Electrophoregrams of the sequences of the antisense strand are shown. (**C**) 14504del mutation in the *ND6* gene. Electrophoregrams of the sequences of the antisense strand are shown. The mutation is present in the tumour lesion but not in the adjacent mucosal tissue.

Hereafter, we focus on nonsynonymous mutations in the *ND* genes. A total of 77 different nonsynonymous SNVs and SNPs, including 3 nonsense mutations, were found (Fig. 2A, Supplementary Tables S1 and S2). The properties of each SNV and SNP are summarized in Supplementary Table S2, including the frequency of appearance in cancer tissues (this study) and the occurrence rate in 672 Japanese patients with Parkinson’s disease, Alzheimer’s disease and type 2 diabetes with or without vascular lesions, in men with or without juvenile obesity, and in centenarians of Gifu and Tokyo (GiiB-JST mtSN database, http://mtSNV.tmig.or.jp/mtSNV/)^15,16^. Other properties included the Grantham value, which indicates a drastic physicochemical amino acid change when it is greater than 50^17^; the evolutionary conservation of the original amino acid residue; disease associations (MITOMAP database, http://www.mitomap.org/foswiki/bin/view/MITOMAP/MutationsCodingControl); the effects of the altered amino acid on the conformation of the ND protein; and the MutPred score (predicted pathogenicity score), which is determined by a set of features reflecting protein structure and its dynamics, the presence of functional residues, the biases of the amino acid sequence, and evolutionary conservation at the substitution site and in its neighbourhood, and which indicates high pathogenicity at values greater than 0.7^18^. In investigating the involvement of pathogenic SNVs and SNPs of the *ND* gene in distant metastasis, we classified the cases into primary tumours without distant metastasis (Group 1) and primary tumours with distant metastases and distant metastatic lesions (Group 2) (Fig. 2A). To examine the correlation between the number of cases with any of the somatic mutations and distant metastasis, we counted the number of cases harbouring one or more somatic mutations in each group (Supplementary Table S2). We avoided duplication of the matched cases in the colon cancer specimens. The results showed no correlation (Table 2). We also included nonsynonymous SNVs and SNPs with Grantham values greater than 50. We excluded the A10398G SNP (Grantham value 58) in the *ND3* gene from analysis because it was clear that the occurrence rate in primary tumours and metastases was simply proportional to the number of cases examined (Supplementary Table S2). Again, we did not find any association (data not shown). We reasoned that this lack of association was because all SNVs and SNPs with Grantham values greater than 50 do not always affect complex I activity. Then, we extracted nonsynonymous SNVs and SNPs that had a high probability of decreasing complex I activity, on the basis of five criteria: whether a given SNV or SNP 1) had a Grantham value > 50, 2) was evolutionarily conserved, 3) induced a conformational change in the ND protein, 4) had reported disease associations, and 5) had a MutPred score > 0.7. We considered SNVs and SNPs that satisfied at least three criteria to be pathogenic, and we used all the criteria for extraction. As a result, we selected 2 missense SNPs, 9 missense SNVs and 3 nonsense mutations (Fig. 2B, Supplementary Table S5). Interestingly, SNVs and SNPs that satisfied the criteria were predominantly (more than 5-fold) localized in the *ND* genes, as compared with the *COII*, *ATP6* and *CYTB* genes per unit length (Table 1). The SNP T3394C and SNVs (T3398C and T12338C) change an evolutionarily highly conserved amino acid and have been reported by at least two independent laboratories to be associated with various mitochondria-related diseases (as secondary mutations), and had very high MutPred scores (Fig. 2B, Supplementary Table S5, MITOMAP database). We detected T3394C in the tumour lesion but not in adjacent normal mucosa in one patient, thus indicating that T3394C is a somatic mutation (Fig. 3A). The SNVs (G3709A, T10363C, C11409T, G13103A and T14138C) also change an evolutionarily highly conserved amino acid (Fig. 3A, Supplementary Figure S2). The SNP C3497T and the SNVs (C3689G, G3709A and G3955A) are likely to result in a conformational change in the mitochondrial matrix side of the ND1 protein, which provides the main contact area with the Q module of the peripheral arm, on the basis of analysis *in silico* using SWISS-MODEL (Fig. 4, Supplementary Table S5). None of the other missense mutations listed in Supplementary Table S2 affected the protein structure. The SNVs (G12813A and G13366A and 14504delA) cause premature termination of the translation of the proteins (Fig. 5, Supplementary Table S5). Interestingly, the frequency of T3394C, T3398C, C3497T and T12338C in tumour tissues was more than twice the frequency in Japanese patients with mtDNA-associated diseases and healthy centenarians in the mtSNP database, thus suggesting that these mutations are more likely to occur in cancer cells than in noncancerous cells. Remarkably, we found a highly statistically significant association between the number of cases harbouring at least one of the selected SNPs and SNVs and distant metastasis in NSCLC cases (*P* = 0.0104 by Chi-square test), and the association was also significant for the combined cases of NSCLC and colon cancer (*P* = 0.0109) (Table 2). A more significant association was observed when we evaluated only the lung adenocarcinoma cases (*P* = 0.0036) and when we evaluated only the combined cases of lung and colon adenocarcinomas (*P* = 0.0018) (Table 2, Supplementary Table S5). The association was significant even by the Chi-square test with Yates’ correction (Table 2). We also analysed the association of SNVs and SNPs that satisfied at least two of the above five criteria with distant metastasis, but we did not find any associations. Finally, there was no correlation between the incidence of the selected SNVs and SNPs and age (<60 *vs* 60 ≦ , *P* = 0.315), gender (male *vs* female, *P* = 0.250), tumour stage (T1/T2 *vs* T3/T4, *P* = 0.465) and lymph node metastasis status (negative *vs* positive, *P* = 0.184) in primary tumours.

**Table 2:** Correlation between the selected SNPs and SNVs of *ND* genes and distant metastasis.

Category	Cancer	No. of cases with the indicated mutation/Total No. of cases		Significance
		Group 1 (Primary w/o distant metastasis)	Group 2 (Primary w/distant metastasis and metastases)	
Somatic mutations (missense and nonsense)	NSCLC	5/44	9/38	ns
	Colon cancer	6/15	4/16	ns
	Total	11/59	13/54	ns
Selected SNPs and SNVs	NSCLC	4/44	12/38	*p* = 0.0104* *p* < 0.05**
	Colon cancer	3/15	5/16	ns
	Total	7/59	17/54	*p* = 0.0109* *p* < 0.05**
	Lung adenocarcinoma	1/22	12/29	*p* = 0.0036* *p* < 0.01**
	Colon adenocarcinoma	2/15	3/9	ns
	Total	3/37	15/38	*p* = 0.0018* *p* < 0.01**

Each case with 2 individual mutations was counted only once. Each matched case was also counted only once.

*Significance was evaluated by Chi-square test.

**Significance was evaluated by Chi-square test with Yates’ correction.

ns: not significant.

## Discussion

The cybrid P29mtB82M cells harbouring the 13885insC mutation in the *ND6* gene showed higher spontaneous metastatic potential than did P29mtP29 cells. A PCR array analysis revealed higher expression levels of metastasis-related genes such as *Mmp11*, *Plaur*, *Ccl7*, *Kras*, *Myc*, *Cd44* and *Vegfa*. MMP11 and Plaur are involved in the degradation of the extracellular matrix^19,20^. Ccl7 attracts monocytes and is associated with the recruitment of tumour-associated macrophages, which enhance tumour malignancy by stimulating tumour angiogenesis, tumour cell invasion, migration, and intravasation, and by suppressing anti-tumour immune responses^21–23^. Kras and Myc increase malignancy^24–26^. CD44 is a cancer stem cell marker associated with metastasis and stress resistance^27^. We focused on VEGF expression and found that it was higher in P29mtB82M cells than in P29mtP29 cells under both normoxic and hypoxic conditions. These increased VEGF levels are clearly responsible for stimulating tumour angiogenesis, thus facilitating tumour cell growth and metastasis. Although PCR array analysis showed that a metastasis suppressor gene, *Mtss1*, was upregulated and that three metastasis enhancer genes, *Pnn* (Pinin), *Lpar6* (lysophosphatidic acid receptor 6) and *Fxdy5* (dysadherin), were downregulated (Fig. 1B), the overall metastatic ability of P29mtB82M cells was remarkably high. We also found upregulation of the key enzymes in the glycolytic pathway, thus suggesting the activation of glycolysis (Warburg effect) in P29mtB82M cells. However, PDK1, which inactivates the mitochondrial gatekeeper PDH, was downregulated, thus suggesting increased generation of acetyl-CoA in P29mtB82M cells. These results indicated that, beyond their enhanced glycolysis, P29mtB82M cells, compared with P29mtP29 cells, utilize succinate dehydrogenase (complex II), which participates in both the TCA cycle and the electron transport chain, thus generating more ATP. Importantly, the expression of HIF-1α was increased in P29mtB82 cells, which may have caused the upregulation of *Vegfa* and glycolytic enzyme genes as well as hypoxia resistance, a potentially important phenotype allowing survival during metastatic process. Collectively, it is highly possible that these phenotypic changes may collaboratively enhance distant metastasis of P29mtB82M cells, although enhanced glycolysis alone is not sufficient to induce metastasis^28^. Because we have previously demonstrated that the 13885insC mutation also enhances the lung-colonizing potential of mouse fibrosarcoma cells^6^, the mutation appears to enhance metastasis regardless of tumour cell type. The exact mechanisms by which the *ND6* mutation modulates the expression of the nuclear genes remain to be examined. However, at least, ROS production is likely to be responsible for the upregulation of HIF-1α because mitochondrial ROS enhance the transcription of the *HIF-1α* gene^29^. It is well known that retrograde and anterograde communication exists between the mitochondrial and nuclear genomes. For example, recent reports demonstrate that mtDNA mutations and SNPs affect the expression of nuclear genes through changes in nuclear DNA methylation^30^ and through the activation of the SIRT/FOXO/SOD2 axis of the mitochondrial unfolded protein response (UPR^mt^)^31^. These mechanisms may also play roles in the changes of metastasis-related gene expressions by the mutation.

Curiously, *ND* gene mutations have mostly been reported to be associated with metastasis in experimental settings^6,7,9,11^. In this study, the predicted pathogenic SNVs and SNPs were also predominantly localized in the *ND* genes, thus suggesting the significance of complex I deficiency in enhancing metastasis. The reason is unknown, but we speculate that the dysfunction of other complexes is less favourable than that of complex I for cancer cells to metastasize. Complex III transfers electrons from both complex I and complex II and is a major site of ROS generation^32,33^. Thus, dysfunction of complex III may result in a more marked enhancement of ROS generation and a more severe reduction of ATP production compared with complex I. Hence, pathogenic mutations in the *CYTB* gene may cause more detrimental consequences of oxidative stress, including apoptosis and necrosis, to cancer cells than pathogenic mutations in the *ND* genes. Dysfunction of complex IV and V probably reduces ATP production considerably. Because dysfunction of complex I may produce a modest level of ROS and ROS enhance cell proliferation and survival at low or moderate levels^34^, it might be favourable for cancer cells to survive, invade and metastasize.

An interesting aspect of this study is that homoplasmic states are less prevalent in metastatic cancer cells than in other cancer cells. Because we used core metastases for the analyses, the possibility of the contamination of surrounding healthy tissues can be excluded. One of the explanations is that an *ND* mutation does not have to be homoplasmic in exerting its pathogenic effect, as demonstrated in mitochondrial diseases^3^. The more pathogenic a mutation is, the more it can be heteroplasmic in cells, although the mutant loads should exceed the pathogenic threshold. The fact that heteroplasmic states are prevalent in metastatic cancer cells would indicate that drastically pathogenic somatic mutations are more prevailing characteristics in metastatic cancer cells than in nonmetastatic cancer cells. Consistent with this possibility, the state of heteroplasmy itself is important for metastasis, as demonstrated in a recent paper that showed that mtDNA heteroplasmy correlates with the invasion of breast cancer cells^31^.

It is reasonable to conclude that not all nonsynonymous SNVs and SNPs in the *ND* genes show pathogenicity with regard to the decreased complex I activity. Therefore, it was not logical to use the total number of nonsynonymous SNVs and SNPs found in clinical samples for the analysis of association with clinicopathological features. In this study, we focused on SNVs and SNPs with a high probability of pathogenicity, on the basis of the Grantham value, evolutionary conservation, effect on protein conformation, disease association, and predicted pathogenicity score. We selected 12 SNVs and 2 SNPs. Evolutionary conservation occurs because mutations of these amino acids impair protein function, and conserved amino acids are the most critical for protein function. The SNP T3394C and the SNVs (T3398C, G3709A, T10363C, C11409T, T12338C, G13103A and T14138C) are mutations of conserved amino acids. The SNPs (T3394C and C3497T) and the SNV T3398C are associated with mitochondrial diseases as secondary mutations. The SNP C3497T and the SNVs (C3689G, G3709A and G3955A) have been suggested to cause the conformational change in the ND1 protein, on the basis of *in silico* analysis using SWISS-MODEL. Notably, the protein structure was specifically changed by mutations at the mitochondrial matrix side, where the Q module docks^1,2^. Therefore, although SWISS-MODEL might not be an accurate predictor of the protein structure, the conformational change at this site of the ND1 protein might affect complex I activity. However, the conformational change occurred exclusively in the coiled region, which must be flexible. Therefore, we are not currently certain of the extent to which these SNVs affect the docking of the Q module onto the ND1 protein. Further studies are required to address this question.

Using the selected SNVs and SNPs in the *ND* genes, we found a clear association between the incidence of these SNVs and SNPs and distant metastasis in the cases of both NSCLC alone and the combination of NSCLC and colon cancer. However, one of the limitations of this study is that the association was obtained on the basis of presumptions about the properties of each of the selected missense SNVs and SNPs, and thus there is no concrete evidence. However, we believe that such a statistically clear association is unlikely to be a haphazard result. In the future, the adequacy of the prediction of pathogenicity used here should be confirmed by examining the influence of each missense mutation in cancer cells on complex I (NADH dehydrogenase) activity and ROS generation. The other limitation is that no clear significance was obtained in the colon cancer cases, although the lack of significance was probably due to the small number of cases included. However, because various *ND* mutations have been shown to stimulate the invasion and metastasis of various types of tumours^7–10^, pathogenic *ND* gene mutations appear to generally influence metastatic ability irrespective of human cancer types.

The complex I subunits are encoded by a total of 44 genes in both the mtDNA and the nuclear DNA^35^. Mutations have been reported in 21 complex I nuclear genes to date, some of which also decrease complex I activity^36^. Therefore, SNVs in the nuclear genes might also influence the metastatic behaviour of human cancer cells.

To the best of our knowledge, no previous epidemiological data have demonstrated a high frequency of distant metastasis in patients with mitochondrial diseases. Perhaps such studies have never been performed. Therefore, it would be interesting to investigate the metastatic risk of cancers in patients with mitochondrial diseases, especially mitochondrial diseases caused by complex I deficiency. It would also be intriguing to investigate the metastatic risk of cancer patients with the selected SNPs, G3394C and C3497T. Further studies on these topics would provide valuable information concerning the prediction and prevention of mtDNA-regulated distant metastasis.

## Methods

### Cells and cell culture

P29mtP29 and P29mtB82M cybrid cells that were reintroduced with P29 wild type mtDNA and mtDNA harbouring a *ND6* 13885insC mutation from mouse high-metastatic B82M fibrosarcoma cells into ρ°P29 cells, respectively^6^, were cultured in Dulbecco’s modified Eagle’s medium (DMEM) containing 10% foetal bovine serum (FBS) and 40 µg/ml gentamicin in a humidified atmosphere with 21% O_2_/5% CO_2_. These cells were free of mycoplasma contamination as tested with an e-Myco Mycoplasma PCR Detection Kit (Cosmo Bio Co Ltd., Tokyo, Japan). Hypoxic culture condition (1% O_2_, 94% N_2_, and 5% CO_2_) was achieved in a humidified automatic O_2_/CO_2_ incubator (WAKENYAKU CO. LTD., Kyoto, Japan). In some experiments, the cells were cultured in a severe hypoxic environment in a BD GasPak EZ Anaerobic Container System (BD Biosciences, Franklin Lakes, NJ, USA).

### Spontaneous metastasis assay

All animal experiments were performed in compliance with the institutional guidelines for the care and use of animals in research. The protocol was approved by the IZUMO Campus Animal Care and Use Committee of Shimane University (Approval no: IZ26-7 and IZ27-37). P29mtP29 and P29mtB82M cells (4 × 10^5^ cells/mouse) were subcutaneously implanted into 6-week-old male C57BL/6 mice (Japan SLC, Shizuoka, Japan) (n = 6-7 mice per group). When the tumour volumes had reached approximately 3 cm^3^, as calculated by the equation tumour volume (V) = (a^2^ × b)/2 (where a is the small diameter, and b is the large diameter), the mice were euthanized by CO_2_ inhalation, and the lungs were removed. The lungs were then fixed in Bouin’s solution, destained in 70% ethanol, and examined for the number of macroscopic lung metastatic nodules. The health of the mice was monitored twice weekly after the tumour cell injection.

### Enzyme-linked immunosorbent assay (ELISA) for VEGF measurement

VEGF released into the culture medium was measured by ELISA. For this purpose, the cells were seeded at a concentration of 1 × 10^6^ cells per well in a 24-well plate (BD Falcon, Franklin Lakes, NJ, USA) in 0.5 ml of DMEM containing 1% FBS. Conditioned medium was harvested after 24 h of cultivation. VEGF concentrations were measured with a Mouse VEGF Quantikine ELISA Kit (MBL).

### PCR array analysis of metastasis-related gene expression

The metastasis-related gene expression profile in P29mtP29 and P29mtB82M cells was examined by using Mouse Tumour Metastasis RT^2^ Profiler PCR Arrays (Qiagen, Venlo, Netherlands) according to the manufacturer’s protocol. For data analysis, fold-changes in each gene expression were calculated using the 2^−ΔΔCt^ method, and house-keeping gene controls (average of *Actb*, *B2m*, *Gapdh*, *Gusb* and *Hsp90ab1*) were used for normalization of the results.

### RNA preparation and quantitative RT-PCR (qRT-PCR)

Total RNA was extracted from P29mtP29 and P29mtB82M cells using TRI reagent (Sigma-Aldrich), and 1 µg of total RNA was reverse-transcribed with ReverTraAce qPCR RT Master Mix (TOYOBO, Osaka, Japan). Quantitative real-time PCR was performed with THUNDERBIRD SYBR qPCR Mix (TOYOBO) in a total volume of 20 µl in a Thermal Cycler Dice Real Time System TP860 (TaKaRa, Shiga, Japan): 95 °C for 1 min followed by 40 cycles of denaturation (95 °C for 15 sec) and extension (60 °C for 1 min). Experiments were performed in triplicate. After amplification, dissociation curve analyses were performed to confirm the amplicon specificity for each PCR run. The relative levels of gene expression in P29mtP29 and P29mtB82M cells were normalized against mouse *Gapdh*. Quantification was performed using the 2^−ΔΔCT^ method. The primer sets are shown in Supplementary Table S6.

### CD31 immunohistochemistry

Vessel density in P29mtP29 and P29mtB82M tumours was determined by staining tissue sections for CD31. For this purpose, surgically removed tumours were immediately embedded and frozen in optimal cutting temperature (OCT) compound. Next, cryostat sections (8-µm thick) were cut and fixed in 4% paraformaldehyde for 10 min, blocked with 1% bovine serum albumin (BSA) in Dulbecco’s phosphate-buffered saline (DPBS) and then incubated with rat anti-mouse monoclonal CD31 antibody (BD Biosciences, 550274, 1:100). After being washed with DPBS, the sections were incubated with Alexa Fluor 594-conjugated goat anti-rat IgG (Invitrogen, Thermo Fisher Scientific, A-11007, 1:300) for 1 h. After counterstaining with 4,6-diamidino-2-phenylindole (DAPI), the sections were observed under a confocal laser scanning microscope (Fluoview FV1000, Olympus, Tokyo, Japan). The pixel values of the CD31-positive areas were calculated for each image to determine the tumour vessel density using the ImageJ software (National Institutes of Health).

### Cell viability assay

P29mtP29 and P29mtB82M cells were incubated for 2 days under severe hypoxic conditions. Cell viability was assayed with a trypan blue dye exclusion test.

### Clinical samples

The study was approved by the Chiba Cancer Center Ethics Committee (Approval no. 21-1) in accordance with the Helsinki declaration and was conducted according to the protocol for the collection of surgically removed specimens. Patients with NSCLC and colon cancer who had undergone surgery from 2002 to 2009 were enrolled in this study (Supplementary Table S1). The patients had given consent for their samples to be used for the evaluation of new potential biomarkers, and informed consent was obtained from all patients. Surgically removed primary tumours and distant metastases (brain metastases for NSCLC, and liver and lung metastases for colon cancer) were stored at −80 °C.

### DNA isolation and sequencing of ND genes

The total DNA of frozen tissue samples was isolated by the phenol/chloroform extraction method followed by ethanol precipitation or QIAamp genomic DNA kits (Qiagen). In some cases, DNA was also extracted from paraffin-embedded tissues with TaKaRa DEXPAT (TAKARA BIO INC., Shiga, Japan) according to the manufacturer’s protocol. The mtDNA genes were amplified by PCR of the extracted DNA. The PCR conditions were set at 94 °C for 1 min followed by 30 cycles of amplification at 94 °C for 30 sec, 53 °C for 30 sec and 72 °C for 1 min, with a final extension at 72 °C for 1 min. PCR products were purified with a QIAquick PCR Purification Kit (Qiagen) or gel-purified and then subjected to direct sequencing. The PCR primers are listed in Supplementary Table S6.

### Bioinformatics analysis of wild-type and mutant ND proteins

The structures of the wild-type and mutant ND proteins were calculated with the SWISS-MODEL Server (Swiss Institute of Bioinformatics, Lausanne, Switzerland, https://swissmodel.expasy.org)^37^.

### Statistical analysis

All data are presented as the mean ± s.d. The statistical significance between data sets was tested with two-tailed Student’s *t*-tests with unpaired analysis. Statistical analyses were also performed using non-parametric Mann-Whitney *U* tests and Chi-square tests with or without Yates’ correction to evaluate the differences in the number of metastases in animal experiments and the associations between *ND* gene mutations and metastasis, respectively. *P* < 0.05 was considered significant.

### Data availability

All data generated or analysed during this study are included in this published article (and its Supplementary Information files).

## Electronic supplementary material

Supplementary Information
