# Supplementary material for: Association of predicted pathogenic mutations in mitochondrial ND genes with distant metastasis in NSCLC and colon cancer
Source: Sci Rep. 2017 Nov 14;7:15535. doi: 10.1038/s41598-017-15592-2 (PMC5686070; doi:10.1038/s41598-017-15592-2)
Supplement: Supplementary file 1 — Supplementary Information [file 41598_2017_15592_MOESM1_ESM.pdf]

Title: Association of predicted pathogenic mutations in mitochondrial *ND* genes with distant metastasis in NSCLC and colon cancer

Authors: Nobuko Koshikawa[1], Miho Akimoto[2*], Jun-Ichi Hayashi[3], Hiroki Nagase[1], Keizo Takenaga[2]

Affiliations:

[1]Laboratory of Cancer Genetics, Chiba Cancer Center Research Institute, 666-2 Nitona, Chuoh-ku, Chiba 260-8717, Japan

[2]Department of Life Science, Shimane University Faculty of Medicine, 89-1 Enya, Izumo, Shimane 693-8501, Japan

[3]University of Tsukuba, 1-1-1 Tennodai, Tsukuba, Ibaraki 305-8572, Japan

Corresponding author: Takenaga K. biokeizo@med.shimane-u.ac.jp

*Present address: Department of Biochemistry, Teikyo University School of Medicine, 2–11-1 Kaga, Itabashi-ku, Tokyo 173–8605, Japan

Supplementary Table S1. Nonsynonymous SNPs and SNVs in the *ND* genes in NSCLC and colon cancer clinical samples.

NSCLC primary tissue (45 cases)

Legend: None (grey cell) | Conserved amino acid substitution | Novel mutation

ID	Age/Sex	Histology	TNM*1	ND1	ND2	ND3	ND4L	ND4	ND5	ND6		COII	ATP6	CYTB
LgCa-150	71/M	Squamous cell carcinoma	T3N2M0		G4705A (M79T)<br>A4824G (T119A)				T12880C (F182L)				A8563G (T13A)<br>C8794T (H90Y)	C14766T (T7I)<br>A15326G (T194A)
LgCa-151	57/M	Squamous cell carcinoma	T2N0M0			A10398G (T114A)		G11696A (V313I)					A8701G (T59A)	C14766T (T7I)<br>A15326G (T194A)
LgCa-152	59/F	Adenocarcinoma	T4N0M0			A10398G (T114A)							A8701G (T59A)	C14766T (T7I)<br>T14979C (I78T)<br>A15326G (T194A)
LgCa-153	64/M	Large cell carcinoma	T4N2M1<br>(Mediastinal LN meta)			A10398G (T114A)		A11084G (T109A)				G8075A (A164T)	A8531G (N2S)<br>A8701G (T59A)	C14766T (T7I)<br>A15326G (T194A)
LgCa-154	76/F	Squamous cell carcinoma	T1N0M0			A10398G (T114A)							A8701G (T59A)<br>T8762C (I79T)	C14766T (T7I)<br>A15326G (T194A)
LgCa-157	63/M	Squamous cell carcinoma	T1N0M0			A10398G (T114A)							A8701G (T59A)	C14766T (T7I)<br>A15326G (T194A)
LgCa-159	59/M	Squamous cell carcinoma	T2N1M0		A4833G (T122A)	A10398G (T114A)			G12622A (V96I)				A8701G (T59A)	C14766T (T7I)<br>A15326G (T194A)
LgCa-161	71/M	Adenocarcinoma	T2N1M0			A10398G (T114A)		A11084G (T109A)					A8701G (T59A)	C14766T (T7I)<br>A15326G (T194A)
LgCa-162	62/M	Large cell carcinoma	T1N0M0	G4048A (D248N)	G5460A(A331T)	T10345C (I96T)<br>T10398G (T114A)			T12811C (Y159H)			G7653A (V90I)	A8701G (T59A)	C14766T (T7I)<br>A15326G (T194A)
LgCa-163	57/M	Adenocarcinoma	T1N0M0			A10398G (T114A)			G13708A (A458T)				A8701G (T59A)	C14766T (T7I)<br>A15326G (T194A)
LgCa-164	37/M	Adenocarcinoma	T1N0M0			A10398G (T114A)		A11084G (T109A)					A8701G (T59A)	C14766T (T7I)<br>A15326G (T194A)
LgCa-165	79/F	Adenocarcinoma	T2N2M0			A10398G (T114A)							A8701G (T59A)	C14766T (T7I)<br>T14979C (I78T)<br>A15326G (T194A)
LgCa-166	71/M	Squamous cell crcinomaa	T4N2M0						A12358G (T8A)					C14766T (T7I)<br>A15326G (T194A)
LgCa-167	56/F	Adenocarcinoma	T1N0M0						A12358G (T8A)					C14766T (T7I)<br>A15326G (T194A)
LgCa-168	61/F	Adenocarcinoma	T1N0M0			A10398G (T114A)			G13477A (A381T)	T14502C (I58V)			A8701G (T59A)	C14766T (T7I)<br>A14870G (I42V)<br>T15071C (Y109H)<br>A15218G (T158A)<br>A15326G (T194A)
LgCa-169	72/M	Squamouse cell carcinoma	T1N1M0	G3391A (G29S)<br>G3709A (A135T)<br>(heteroplasmy or mixture)		A10398G (T114A)							A8701G (T59A)	C14766T (T7I)<br>A15326G (T194A)
LgCa-173	74/M	Squamouse cell carcinoma	T3N0M0		T5344C (F292S)<br>(heteroplasmy or mixture)<br>G5460A (A331T)	T10345C (I96T)<br>T10398G (T114A)			T12811C (Y159H)<br>G13103A (G256E)<br>(heteroplasmy or mixture)		A case with two selected mutations	G7853A (V90I)	A8701G (T59A)	C14766T (T7I)<br>G14804A (D20N)<br>A15326G (T194A)
LgCa-174	59/M	Adenocarcinoma	T2N1M0		G4704A (M79L)<br>A4824G (T119A)								A8563G (T13A)<br>C8794T (H90Y)	C14766T (T7I)<br>A15326G (T194A)
LgCa-175	46/F	Adenocarcinoma	T1N0M0										T9101G (I192S)<br>(homoplasmy)	C14766T (T7I)<br>A15326G (T194A)
LgCa-176	80/M	Squamouse cell carcinoma	T1N0M0		T4705C (M79T)<br>A4732G (N88S)		T10609C (M47T)		G12406A (V24I)<br>G13928C (S531T)					C14766T (T7I)<br>A15326G (T194A)
LgCa-177	43/F	Squamouse cell carcinoma	T1N0M0			A10398G (T114A)							A8701G (T59A)	C14766T (T7I)<br>A15326G (T194A)
LgCa-178	58/M	Squamouse cell carcinoma	T1N1M0	T4216C (Y304H)	A5301G (I278V)	A10398G (T114A)			A13105G (I257V)				A8701G (T59A)	C14766T (T7I)<br>A14927G (T61A)<br>A15326G (T194A)

LgCa-179	54/F	Adenocarcinoma	T1N0M0			A10398G (T114A)							C14766T (T7I)<br>A15326G (T194A)
LgCa-180	68/M	Adenocarcinoma	T2N1M0		A4824G (T119A)				T12880C (F182L)			A8563G (T13A)<br>C8794T (H90Y)	C14766T (T7I)<br>A15326G (T194A)
LgCa-181	64/M	Squamouse cell carcinoma	T1N2M0			A10398G (T114A)						G8584A (A20T)<br>A8701G (T59A)	C14766T (T7I)<br>A15326G (T194A)
LgCa-182	46/M	Adenocarcinoma	T1N2M0			A10398G (T114A)		A11084G (T109A)				T8602C (F26L)<br>A8701G (T59A)	C14766T (T7I)<br>A15326G (T194A)
lgCa-183	67/M	Squamouse cell carcinoma	T2N0M0										C14766T (T7I)<br>T14798C (F18L)<br>A15236G (I164V)<br>A15326G (T194A)
LgCa-184	52/M	Pleomorphic carcinoma	T3N0M0						A12358G (T8A)				C14766T (T7I)<br>A15326G (T194A)
LgCa-187	73/M	Adenocarcinoma	T1N0M0		T4705C (M79T)<br>A4732G (N88S)		T10609C (M47T)		G12406A (V24I)<br>G13928C (S531T)				C14766T (T7I)<br>A15326G (T194A)
LgCa-188	59/M	Squamouse cell carcinoma	T1N0M0			A10398G (T114A)						A8701G (T59A)	C14766T (T7I)<br>T14979C (I78T)<br>A15326G (T194A)
LgCa-189	73/M	Squamouse cell carcinoma	T1N1M0		A5301G (I278V)	A10398G (T114A)			A13651C (T439P)	T14319C (N119D)		A8701G (T59A)	C14766T (T7I)<br>A15326G (T194A)
LgCa-190	62/M	Adenocarcinoma	T2N1M0					G11016A (S86N)	A13183G (I283V)				C14766T (T7I)<br>A15326G (T194A)
LgCa-191	50/M	Adenocarcinoma	T2N2M0			A10398G (T114A)			A12361G (T9A)			G8584A (A20T)	C14766T (T7I)<br>A15326G (T194A)
LgCa-192	54/F	Adenocarcinoma	T4N0M0		G4705A (M79T)<br>A4824G (T119A)							A8563G (T13A)<br>C8794T (H90Y)	C14766T (T7I)<br>A15326G (T194A)
LgCa-193	76/M	Squamouse cell carcinoma	T1N0M0			A10398G (T114A)						A8701G (T59A)	C14766T (T7I)<br>A15326G (T194A)
LgCa-194	41/M	Adenocarcinoma	T2N2M0		A5301G (I278V)	A10398G (T114A)						A8701G (T59A)	C14766T (T7I)<br>A15326G (T194A)
LgCa-195	66/M	Bronchioalveolar carcinoma	T1N0M0									A8701G (T59A)	C14766T (T7I)<br>A15326G (T194A)
LgCa-196	64/M	Adenocarcinoma	T1N0M0	C3497T (A64V)	A4704C (M79L)								C14766T (T7I)<br>A15326G (T194A)
LgCa-197	59/M	Bronchioloalveolar carcinoma	T1N0M0	T3394C (Y30H)	G4491A (V8I)	G10290A (A78T)<br>(heteroplasmy or mixture)<br>A10389G (T114T)		G11963A (V402I)				A8701G (T59A)	C14766T (T7I)<br>A15326G (T194A)
LgCa-198	35/M	Squamous cell carcinoma	T?N1M0			A10398G (T114A)		A11084G (T109A)				A8701G (T59A)	C14766T (T7I)<br>A15326G (T194A)
LgCa-199	63/M	Adenocarcinoma	T1N1M0						A12358G (T8A)				C14766T (T7I)<br>A15326G (T194A)
LgCa-200	58/F	Adenocarcinoma	T1N0M0	G4048A (D248N)	G5460A (A331T)	T10345C (I96T)<br>T10389G (T114T)			T12811C (Y159H)		G7853A (V90I)	A8701G (T59A)	C14766T (T7I)<br>A15326G (T194A)
LgCa-201	64/M	Adenocarcinoma	T1N0M0			A10398G (T114A)			A12361G (T9A)			G8584A (A20T)	C14766T (T7I)<br>G14985A (R80H)<br>A15326G (T194A)
LgCa-202	76/M	Adenocarcinoma	T1N0M0			A10398G (T114A)						A8701G (T59A)	C14766T (T7I)<br>A15326G (T194A)
LgCa-205	74/F	Squamous cell carcinoma	T1N1M0			A10398G (T114A)		G11696A (V313I)				A8701G (T59A)	C14766T (T7I)<br>A15326G (T194A)

*1 TNM (Tumour/Node/Metastasis) clasification for staging of cancer.

NSCLC metastases (37 cases)

None | Conserved amino acid substitution | Novel mutation

ID	Age/Sex	Histology	Metastatic site	ND1	ND2	ND3	ND4L	ND4	ND5	ND6	COII	ATP6	CYTB
LuAdBrM-1	–	Adenocarcinoma	Brain			A10398G (T114A)		A11084G (T109A)	C12813A (Y159STOP)<br>(heteroplasmy or mixture)			A8701G (T59A)	C14766T (T7I)<br>A15326G (T194A)

LuAdBrM-2	64/M	Adenocarcinoma	Brain	C3497T (A64V)									C14766T (T7I) A15326G (T194A)
LuAdBrM-3	–	Adenocarcinoma	Brain			A10398G (T114A)		A11084G (T109A)				A8701G (T59A)	C14766T (T7I) A15326G (T194A)
LuAdBrM-4	–	Adenocarcinoma	Brain		A4824G (T119A)	T10363C (L102P) (heteroplasmy or mixture)						A8563G (T13A) C8794T (H90Y)	C14766T (T7I) A15326G (T194A)
LuAdBrM-5	79/M	Adenocarcinoma	Brain	T3394C (Y30H) [T3394C (Y30H]	G4491A (V8I)	A10398G (T114A)		G11963A (V402I)				A8701G (T59A)	C14766T (T7I) A15326G (T194A)
LuAdBrM-6	–	Adenocarcinoma	Brain	C3689G (A128G) (heteroplasmy or mixture)		T10084C (I9T)			A12358G (T8A)				C14766T (T7I) A15326G (T194A)
LuAdBrM-7	49/M	Adenocarcinoma	Brain		T4612C (M48T) A5127G (N220D)			G11969A (A404T) C11151T (A131V)	A13105G (I257V)				C14766T (T7I) A15326G (T194A)
LuAdBrM-8	70/M	Adenocarcinoma	Brain	T3394C (Y30H) [T3394C (Y30H)]	G4491A (V8I)	A10398G (T114A)		G11963A (V402I)				A8701G (T59A)	C14766T (T7I) A15326G (T194A)
LuAdBrM-9	–	Adenocarcinoma	Brain	C3497T (A64V)				A11930G (I391V) (homoplasmy)	A12361G (T9A)				C14766T (T7I) A15326G (T194A)
LuAdBrM-10	51/M	Adenocarcinoma	Brain		C5178A (L237M)	A10398G (T114A)			A14053G (T573A)			A8701G (T59A)	C14766T (T7I) A15326G (T194A)
LuAdBrM-11	–	Adenocarcinoma	Brain			A10398G (T114A)			G13135A (A267T)	T14502C (I58V) C14347T (V113M) (homoplasmy)		A8701G (T59A)	C14766T (T7I) T15071C (Y109H) A15326G (T194A)
LuAdBrM-12	–	Adenocarcinoma	Brain			A10398G (T114A)						G8584A (A20T)	C14766T (T7I) A15326G (T194A)
LuAdBrM-13	46/M	Adenocarcinoma	Brain	T3394C (Y30H) [T3394C (Y30H)]		A10398G (T114A)							C14766T (T7I) A15326G (T194A)
LuAdBrM-14	–	Adenocarcinoma	Brain						A12358G (T8A)				C14766T (T7I) A15326G (T194A)
LuAdBrM-15	50/M	Adenocarcinoma	Brain			A10398G (T114A)		A11084G (T109A)	T13768C (F478L)			A8701G (T59A)	C14766T (T7I) A15326G (T194A)
LuAdBrM-16	69/M	Adenocarcinoma	Brain		C5178A (L237M)	A10398G (T114A)							C14766T (T7I) A15326G (T194A)
LuAdBrM-17	50/F	Adenocarcinoma	Brain			A10398G (T114A)			A12361G (T9A)			G8584A (A20T)	C14766T (T7I) A15326G (T194A)
LuAdBrM-18	–	Adenocarcinoma	Brain		A4824G (T119A)			C11409T (P217L) (heteroplasmy or mixture)		A14193G (F161L)		A8563G (T13A) C8794T (H90Y)	C14766T (T7I) A15326G (T194A)
LuAdBrM-19	47/M	Adenocarcinoma	Brain			A10398G (T114A)			A12361G (T9A)			G8584A (A20T) A8894T (N123I)	C14766T (T7I) A14750G (T2A) A15326G (T194A)
LuAdBrM-20	70/M	Adenocarcinoma	Brain	G4048A (D248N)	G5460A (A331T)	T10345C (I96T) A10389G (T114A)			T12811C (Y159H)		G7853A (V90I)	A8701G (T59A)	C14766T (T7I) A15326G (T194A)
LuAdBrM-22	72/M	Adenocarcinoma	Brain		C5178A (L237M)	A10398G (T114A)						A8701G (T59A)	C14766T (T7I) A15326G (T194A)
LuAdBrM-23	–	Adenocarcinoma	Brain		C5178A (L237M)	A10398G (T114A)						A8701G (T59A) T8762C (I79T)	C14766T (T7I) A15326G (T194A)
LuAdBrM-24	68/F	Adenocarcinoma	Brain			A10398G (T114A)		A11084G (T109A)				A8701G (T59A)	C14766T (T7I) A15326G (T194A)
LuAdBrM-25	74/F	Adenocarcinoma	Brain		G4705A (M79T) A4732G (N88S)		T10609C (M47T)		G12406A (V24I) G13928C (S531T)				C14766T (T7I) A15326G (T194A)
LuPoBrM-1	–	Adenocarcinoma	Brain	C3497T (A64V)	A4704C (M79L)								C14766T (T7I) A15326G (T194A)
LuPoBrM-2	–	Adenoarcinoma	Brain	C3497T (A64V)					T13879C (S515P)	G14162A (A171V)			C14766T (T7I) A15326G (T194A)
LuPoBrM-3	–	Adenoarcinoma	Brain		A4824G (T119A) T5361C (Y298H) (heteroplasmy or mixture)							C8794T (H90Y)	C14766T (T7I) A15326G (T194A)

LuSQBrM-1	–	Squamous cell carcinoma	Brain		T5442C (F325L)								A8701G (T59A)	C14766T (T7I)<br>A15326G (T194A)
LuSQBrM-2	62/F	Squamous cell carcinoma	Brain			A10398G (T114A)							A8701G (T59A)	C14766T (T7I)<br>T14979C (I78T)<br>A15326G (T194A)
LuSQBrM-3	63/M	Squamous cell carcinoma	Brain			A10398G (T114A)							A8701G (T59A)	C14766T (T7I)<br>A15326G (T194A)
LuSQBrM-4	65/M	Squamous cell carcinoma	Brain			A10398G (T114A)			A13651G (T439A)				A8701G (T59A)	C14766T (T7I)<br>T14979C (I79T)<br>A15326G (T194A)
LuSQBrM-5	79/M	Squamous cell carcinoma	Brain		T4705C (M79T)	A10398G (T114A)			G12406A (V24I)<br>G13928C (S531T)					C14766T (T7I)<br>A15326G (T194A)
LuSQBrM-6	–	Squamous cell carcinoma	Brain									G7664A (A27T)		C14766T (T7I)<br>A15236G (I164V)<br>A15326G (T194A)
LuSQBrM-7	–	Squamous cell carcinoma	Brain	C3497T (A64V)				A11930G (I391V)<br>(homoplasmy)						C14766T (T7I)<br>A15326G (T194A)
LuSQBrM-8	–	Squamous cell carcinoma	Brain			A10398G (T114A)				T14318C (N119S)		G7598A (A5T)	G8584A (A20T)<br>A8701G (T59A)<br>C9099A (I191M)	C14766T (T7I)<br>A15326G (T194A)
LuSqBrM-9<br>(LgCa-194)	41/M	Adenocarcinoma	Brain		C5178A (L237M)<br>A5301G (I278V)									C14766T (T7I)<br>A15326G (T194A)
LuAdBrM-26	54/F	Adenocarcinoma	Brain				T10609C (M47T)		G12406A (V24I)<br>G13928C (S531T)					C14766T (T7I)<br>A15326G (T194A)

Colon cancer primary tissue (22 cases)

None | Conserved amino acid substitution | Novel mutation

ID	Age/Sex	Histology	TNM*1	ND1	ND2	ND3	ND4L	ND4	ND5	ND6		COII	ATP6	CYTB
ColoCa-1	69/M	Adenocarcinoma	T3N1M1		A4824G (T119A)								A8563G (T13A)<br>C8794T (H90Y)	C14766T (T7I)<br>A15326G (T194A)
ColoCa-2	65/M	Adenocarcinoma	T3N2M0		A4833G (T122A)	A10398G (T114A)			G12503A (C56Y)<br>(heteroplasmy or mixture)				A8701G (T59A)	C14766T (T7I)<br>G15323A (A193T)<br>A15326G (T194A)<br>G15497A (G241S)<br>A15860G (I372V)
ColoCa-3	71/M	Adenocarcinoma	T3N1M0		A5301G (I278V)	A10398G (T114A)		A12026G (I426V)	C14003T (T557I)			G7616A (D11N)<br>(homoplasmy)	A8701G (T59A)	C14766T (T7I)<br>G15314A (A190T)<br>A15326G (T194A)
ColoCa-4	50/M	Adenocarcinoma	T4N4M0		T4705C (M79T)<br>A4732G (N88S)		T10609C (M47T)		G12406A (V24I)<br>G13928C (S531T)					C14766T (T7I)<br>A15326G (T194A)
ColoCa-5	67/M	Adenocarcinoma	T4N4M0											C14766T (T7I)<br>A15326G (T194A)
ColoCa-6	67/F	Adenocarcinoma	P(T?N0M1)<br>(Liver meta)			A10398G (T114A)							A8701G (T59A)	C14766T (T7I)<br>A15326G (T194A)
ColoCa-7	52/F	Adenocarcinoma	T4N2M1<br>(Liver meta)	T3394C (Y30H)<br>(somatic mutation)		A10398G (T114A)								C14766T (T7I)<br>A15326G (T194A)
ColoCa-8	61/M	Adenocarcinoma	T3N0M0		A4824G (T119A)				A14062G (I576V)<br>(homoplasmy)				C8794T (H90Y)	C14766T (T7I)<br>A15326G (T194A)
ColoCa-9	66/M	Adenocarcinoma	T2N0M0		A5301G (I278V)	A10398G (T114A)							A8701G (T59A)	C14766T (T7I)<br>A15326G (T194A)
ColoCa-10	64/M	Adenocarcinoma	T3N0M0			A10398G (T114A)		G11696A (V313I)					A8701G (T59A)	C14766T (T7I)<br>A15326G (T194A)
ColoCa-11	61/F	Adenocarcinoma	T3N1M0			A10398G (T114A)							A8701G (T59A)	C14766T (T7I)<br>A15326G (T194A)
ColoCa-12	53/F	Adenocarcinoma	T3N1M0		T5442C (F161L)	A10398G (T114A)		A11510G (N251D)<br>(heteroplasmy or mixture)					A8701G (T59A)	C14766T (T7I)<br>A15326G (T194A)
ColoCa-13	66/M	Adenocarcinoma	T3N1M1<br>(Liver meta)	G3736A (V144I)	T4705C (M79T)<br>A4732G (N88S)	A10398G (T114A)	T10609C (M47T)		G12406A (V24I)<br>G13928C (S531T)		Matched case 1			C14766T (T7I)<br>A15326G (T194A)

ColoCa-14	74/F	Adenocarcinoma	T1N0M0									G7859A (D92N)		C14766T (T7I)<br>A15326G (T194A)<br>C15535T (N261D)
ColoCa-15	82/F	Adenocarcinoma	T1N0M0	G4048A (D248N)	G5460A(A331T)	T10345C (I96T)<br>A10389G (T114A)			T12811C (Y159H)<br>G13366A (G344STOP)<br>(Heterogeneous or Heteroplasmy)	14504 delA (57STOP)<br>(homoplasmy)	A case with two selected SNVs	G7853A (V90I)	A8701G (T59A)	C14766T (T7I)<br>A15326G (T194A)
ColoCa-16	66/M	Neuroendocrine	T1N1M0	T3398C (M31T)		A10398G (T114A)							A8701G (T59A)	C14766T (T7I)<br>A15326G (T194A)
ColoCa-17	75/F	Adenocarcinoma	T1N0M0	T4067C (L254P)<br>(Heterogeneous or Heteroplasmy)					T12338C (M1T)<br>G13708A (A458T)<br>G13928C (S531T)					C14766T (T7I)<br>A15326G (T194A)
ColoCa-18	54/F	Adenocarcinoma	T4N1M1<br>(Liver meta)			A10398G (T114A)		A11084G (T109A)	G12454A (V60I)				A8701G (T59A)	C14766T (T7I)<br>A15218G (T158A)<br>A15326G (T194A)
ColoCa-19	56/M	Adenocarcinoma	T3N0M0			A10398G (T114A)							A8701G (T59A)	C14766T (T7I)<br>T14979C (I78T)<br>A15326G (T194A)
ColoCa-20	62/M	Adenocarcinoma	T1N0M0											C14766T (T7I)<br>A15326G (T194A)
ColoCa-21	63/M	Adenocarcinoma	T?N?M1<br>(Liver meta)			A10398G (T114A)		A11084G (T109A)					A8701G (T59A)	C14766T (T7I)<br>T14934C (F63S)<br>(homoplasmy)<br>A15326G (T194A)
ColoCa-22	67/M	Adenocarcinoma	T4N1M1<br>(Liver meta)		T5418C (F317L)			C12135T (S459F)	A13942G (T536A)		Matched case 2	T8223C (L213S)<br>(Heterogeneous or Heteroplasmy)	A8860G (T112A)	C14766T (T7I)<br>G15254A (V170M)<br>(heteroplasmy or mixture)<br>A15326G (T194A)

*1 TNM (Tumour/Node/Metastasis) clasification for staging of cancer.

Colon cancer metastases (11 cases)

None | **Conserved amino acid substitution** | **Novel mutation**

ID	Age/Sex	Histology	Metastasis site	ND1	ND2	ND3	ND4L	ND4	ND5	ND6		COII	ATP6	CYTB
CoAdBrM-1	–	Adenocarcinoma	Brain	C3689G (A128G)<br>(homoplasmy)		A10398G (T114A)	T10609C (M47T)		G12406A (V24I)<br>G13759A (A475T)<br>G13928C (S531T)				G9053A (S176N)	C14766T (T7I)<br>A15326G (T194A)<br>A15363G (N206S)
ColonLivM-1	–	-	Liver			A10398G (T114A)		A11084G (T109A)					A8701G (T59A)	C14766T (T7I)<br>A15326G (T194A)
ColonLivM-2	–	-	Liver		C5178A (L237M)	A10398G (T114A)							A8701G (T59A)	C14766T (T7I)<br>A15326G (T194A)
ColonLivM-3	–	-	Liver		C5178A (L237M)								A8701G (T59A)	C14766T (T7I)<br>T14979C (I78T)<br>A15326G (T194A)
ColonLivM-4	–	-	Liver	G3955A (A217T)<br>(heteroplasmy or mixture)		A10398G (T114A)		G11016A (S86N)	A13183G (I283V)					C14766T (T7I)<br>A15326G (T194A)
ColonLivM-5	–	-	Liver	C3497T (A64V)	T4833G (T122A)				T14138C (L601P)<br>(heteroplasmy or mixture)		A case with a selected SNP and a SNV		A8701G (T59A)	C14766T (T7I)<br>A15326G (T194A)
ColonLivM-6	–	-	Liver						T13879C (S515P)	G14162A (A171V)				C14766T (T7I)<br>A15326G (T194A)
ColoLivM-8<br>(ColoCa-22)	67/M	Adenocarcinoma	Liver		T5418C (F317L)			C12135T (S459F)	A13942G (T536A)		Matched case 2	T8223C (L213S)<br>(homoplasmy)	A8860G (T112A)	C14766T (T7I)<br>G15254A (V170M)<br>(heteroplasmy or mixture)<br>A15326G (T194A)
ColoCa-13LivM	66/M	Adenocarcinoma	Liver	G3736A (V144I)	T4705C (M79T)<br>A4732G (N88S)	A10398G (T114A)	T10609C (M47T)		G12358A (T8A)<br>G12406A (V24I)<br>G13928C (S531T)		Matched case 1			C14766T (T7I)<br>A15326G (T194A)
ColonLuM-1	80/M	-	Lung			A10398G (T114A)						G7664A (A27T)		C14766T (T7I)<br>A15236G (I164V)<br>A15326G (T194A)<br>A15535T (N261D)
ColonLuM-2<br>(LgCa203)	71/F	Adenocarcinoma	Lung			T10084C (I9T)<br>A10398G (T114A)								C14766T (T7I)<br>A15326G (T194A)

Supplementary Table S2. Nonsynonymous SNPs and SNVs in the *ND* genes observed in NSCLC and colon cancer clinical samples.

Novel mutation

SNP/Mutation	Amino acid substitution	Homoplasmy or heteroplasmy	Frequency T (this study) (%)	Frequency C (mtSNP-Japan)*1 (%)	Frequency (T/C)*2	Grantham value	Evolutionary conservation of the original amino acid	Conformational change	Reported disease association*3	MutPred score*4	No. of cases detected			
											NSCLC		Colon Cancer	
											Primary (N=45)	Metastases (N=37)	Primary (N=22)	Metastases (N=11)
ND1														
G3391A	G29S	Homoplasmy	1/115 (0.87%)	10/672 (1.49%)	0.58	56			LHON (putative)	0.862	1	0	0	0
T3394C	Y30H	Homoplasmy	5/115 (4.35%)	14/672 (2.08%)	2.09	83	Conserved		LHON / Diabetes / CPTdeficiency / high altitude adaptation	0.783	1	3	1 w/ liver meta	0
T3398C	M31T	Homoplasmy	1/115 (0.87%)	2/672 (0.30%)	2.9	81	Conserved		DMDF+HCM / GDM / possibly LVNC cardiomyopathy-associated	0.751	0	0	1	0
C3497T	A64V	Homoplasmy	7/115 (6.09%)	20/672 (2.98%)	2.04	64		Yes	LHON	0.413	1	5	0	1
C3689G	A128G	Heteroplasmy or Heterogeneous	2/115 (1.74%)	0	-	60		Yes		0.747	0	1	0	1
G3709A	A135T	Heteroplasmy or Heterogeneous	1/115 (0.87%)	0	-	58	Conserved	Yes		0.774	1	0	0	0
G3736A	V144I	Homoplasmy	2/115 (1.74%)	1/672 (0.15%)	11.6	29	Conserved		LHON	0.661	0	0	1 w/ liver meta (matched case 1)	1 (matched case 1)
G3955A	A217T	Heteroplasmy or Heterogeneous	1/115 (0.87%)	0	-	58		Yes		0.736	0	0	0	1
G4048A	D248N	Homoplasmy	4/115 (3.48%)	32/672 (4.76%)	0.73	23				0.37	2	1	1	0
T4067C	L254P	Heteroplasmy or Heterogeneous	1/115 (0.87%)	0	-	98				0.804	0	0	1	0
T4216C	Y304H	Homoplasmy	1/115 (0.87%)	2/672 (0.30%)	2.9	83	(Haplogroup JT)		LHON / Insulin Resistance /possible adaptive high altitude variant	0.611	1	0	0	0
ND2														
G4491A	V8I	Homoplasmy	3/115 (2.61%)	11/672 (1.64%)	1.59	29				0.439	1	2	0	0
T4612C	M48T	Homoplasmy	1/115 (0.87%)	3/672 (0.45%)	2.22	81				0.602	0	1	0	0
A4704C	M79L	Homoplasmy	3/115 (2.61%)	1/672 (0.15%)	17.4	15				0.686	2	1	0	0
T4705C	M79T	Homoplasmy	9/115 (7.83%)	9/672 (1.34%)	5.98	81				0.7	4	2	2 (1 w/ liver meta)	1
A4732G	N88S	Homoplasmy	6/115 (5.22%)	19/672 (2.83%)	1.84	46				0.292	2	1	2	1
A4824G	T119A	Homoplasmy	9/115 (7.83%)	50/672 (7.44%)	1.05	58	(Haplogroup A)			0.368	4	3	2	0
A4833G	T122A	Homoplasmy	3/115 (2.61%)	58/672 (8.63%)	0.3	58	(Haplogroup G)		Diabetes helper mutation/AD/PD	0.488	1	0	1	1
A5127G	N220D	Homoplasmy	1/115 (0.87%)	3/672 (0.45%)	1.93	23				0.528	0	1	0	0

C5178A	L237M	Homoplasmy	7/115 (6.09%)	261/673 (38.84%)	0.16	15	(Haplogroup D)		Longevity/Extraversion MI /AMS protection/ blood iron metabolism	0.26	0	5	0	2
A5301G	I278V	Homoplasmy	6/115 (5.22%)	29/672 (4.32%)	1.21	29				0.311	3	1	2	0
T5344C	F292S	Heteroplasmy or Heterogeneous	1/115 (0.87%)	0	-	22	Conserved			0.59	1	0	0	0
T5361C	Y298H	Heteroplasmy or Heterogeneous	1/115 (0.87%)	0	-	83	Conserved			0.685	0	1	0	0
T5418C	F317L	Homoplasmy	2/115 (1.74%)	0	-	22				0.612	0	0	1 w/ liver meta	1
T5442C	F325L	Homoplasmy	2/115 (1.74%)	4/672 (0.60%)	2.9	22				0.437	0	1	1	0
G5460A	A331T	Homoplasmy	5/115 (4.35%)	38/672 (5.65%)	0.77	58			PD/AD (controversial)	0.505	3	1	1	0
**ND3**														
T10084C	I9T	Homoplasmy	2/115 (1.74%)	11/672 (1.64%)	1.06	89				0.414	0	1	0	1
G10290A	A78T	Heteroplasmy or Heterogeneous	1/115 (0.87%)	0	-	58				0.669	1	0	0	0
T10345C	I96T	Homoplasmy	5/115 (4.35%)	29/672 (4.32%)	1	89				0.398	3	1	1	0
T10363C	L102P	Heteroplasmy or Heterogeneous	1/115 (0.87%)	0	-	98	Conserved			0.746	0	1	0	0
A10398G	T114A	Homoplasmy	67/115 (58.26%)	478/672 (71.13%)	0.82	58	(Haplogroup IJK)		PD protective factor / longevity / altered cell pH / metabolic syndrome / breast cancer risk	0.17	28	20	13	7
**ND4L**														
T10609C	M47T	Homoplasmy	8/115 (6.96%)	35/672 (5.21%)	1.34	81				0.481	2	2	2 (1 w/ liver meta)	2
**ND4**														
G11016A	S86N	Homoplasmy	2/115 (1.74%)	19/672 (2.83%)	0.62	46				0.303	1	0	0	1
A11084G	T109A	Homoplasmy	12/115 (10.43%)	38/672 (5.65%)	1.85	58			AD/PD/MELAS (controvertial)	0.482	5 (1 w/ mediastinal LN meta)	4	2 w/ liver meta	1
C11151T	A131V	Homoplasmy	1/115 (0.90%)	5/672 (0.74%)	1.22	64				0.577	0	1	0	0
C11409T	P217L	Heteroplasmy or Heterogeneous	1/115 (0.90%)	0	-	98	Conserved			0.83	0	1	0	0
A11510G	N251D	Heteroplasmy or Heterogeneous	1/115 (0.90%)	0	-	23				0.364	0	0	1	0
G11696A	V313I	Homoplasmy	3/115 (2.61%)	5/672 (0.74%)	3.53	29			LHON / LDYT / DEAF / hypertension helper mutation	0.581	2	0	1	0
A11930G	I391V	Homoplasmy	2/115 (1.74%)	0	-	29				0.553	0	2	0	0
G11963A	V402I	Homoplasmy	3/115 (2.61%)	7/672 (1.04%)	2.51	29				0.369	1	2	0	0
G11969A	A404T	Homoplasmy	1/115 (0.90%)	4/672 (0.60%)	1.5	58				0.639	0	1	0	0

A12026G	I423V	Homoplasmy	1/115 (0.90%)	17/672 (2.53%)	0.36	29			DM	0.277	0	0	1	0
C12135T	S459F	Homoplasmy	2/115 (1.74%)	1/672 (0.15%)	11.6	155				0.319	0	0	1 w/ liver meta (matched case 2)	1 (matched case 2)
**ND5**														
T12338C	M1T	Homoplasmy	1/115 (0.90%)	2/672 (0.30%)	3	81	Conserved		DEAF1555 increased penetrance / LHON	0.966	0	0	1	0
A12358G	T8A	Homoplasmy	7/115 (6.09%)	40/672 (5.95%)	0.88	58				0.265	4	2	0	1
A12361G	T9A	Homoplasmy	5/115 (4.35%)	25/672 (3.72%)	1.02	58				0.249	2	3	0	0
G12406A	V24I	Homoplasmy	9/115 (7.83%)	39/672 (5.80%)	1.35	29				0.251	2	3	2	2
G12454 A	V40I	Homoplasmy	1/115 (0.90%)	0	-	29				0.528	0	0	1 w/ liver meta	0
G12503A	C56Y	Heteroplasmy or Heterogeneous	1/115 (0.90%)	0	-	194				0.399	0	0	1	0
G12622A	V96I	Homoplasmy	1/115 (0.90%)	1/672 (0.15%)	6	29				0.403	1	0	0	0
T12811C	Y159H	Homoplasmy	5/115 (4.35%)	29/672 (4.32%)	1.01	83			Possible LHON factor	0.587	3	1	1	0
C12813A	Y159STOP	Heteroplasmy or Heterogeneous	1/115 (0.90%)	0	-	-		Truncated		-	0	1	0	0
T12880C	F182L	Homoplasmy	2/115 (1.74%)	22/672 (3.27%)	0.53	22				0.635	2	0	0	0
G13103A	G256E	Heteroplasmy or Heterogeneous	1/115 (0.90%)	0	-	98	Conserved			0.834	1	0	0	0
A13105G	I257V	Homoplasmy	2/115 (1.74%)	7/672 (1.04%)	1.67	29				0.35	1	1	0	0
G13135A	A267T	Homoplasmy	1/115 (0.90%)	7/672 (1.04%)	0.87	58				0.452	0	1	0	0
A13183G	I283V	Homoplasmy	1/115 (0.90%)	18/672 (2.68%)	0.34	29				0.48	1	0	0	1
G13366A	G344STOP	Heteroplasmy or Heterogeneous	1/115 (0.90%)	0	-	-		Truncated		-	0	0	1	0
G13477A	A381T	Homoplasmy	1/115 (0.90%)	2/672 (0.30%)	3	58				0.601	1	0	0	0
A13651C	T439P	Homoplasmy	1/115 (0.90%)	11/672 (1.64%)	0.55	38				-	1	1	0	0
G13708A	A458T	Homoplasmy	2/115 (1.74%)	19/672 (2.83%)	0.78	58	(Haplogroup J)		LHON / Increased MS risk / higher freq in PD-ADS	0.409	1	0	1	0
G13759A	A475T	Homoplasmy	1/115 (0.90%)	15/672 (2.23%)	0.4	58				0.556	0	0	0	1
T13768C	F478L	Homoplasmy	1/115 (0.90%)	3/672 (0.45%)	2	22				0.572	0	1	0	0
T13879C	S515P	Homoplasmy	1/115 (0.90%)	5/672 (0.74%)	1.22	74				0.426	0	1	0	1
G13928C	S531T	Homoplasmy	10/115 (8.70%)	2/672 (0.30%)	29	58	(Haplogroup F)			0.321	2	3	3	2
A13942G	T536A	Homoplasmy	2/115 (1.74%)	2/672 (0.30%)	5.8	58				0.438	0	0	1	1
C14003T	T556I	Homoplasmy	1/115 (0.90%)	1/672 (0.15%)	6	89				0.533	0	0	1	0

A14053G	T573A	Homoplasmy	1/115 (0.90%)	4/672 (0.60%)	1.5	58				0.409	0	1	0	0
A14062G	I576V	Homoplasmy	1/115 (0.90%)	0	-	29				0.421	0	0	1	0
T14138C	L601P	Heteroplasmy or Heterogeneous	1/115 (0.90%)	0	-	98	Conserved			0.762	0	0	0	1
**ND6**														
G14162A	A171V	Homoplasmy	2/115 (1.74%)	4/672 (0.60%)	2.9	64				0.597	0	1	0	1
A14193G	F161L	Homoplasmy	1/115 (0.87%)	0	-	22				0.653	0	1	0	0
T14318C	N119S	Homoplasmy	1/115 (0.87%)	2/672 (0.30%)	2.9	46				0.437	0	1	0	0
T14319C	N119D	Homoplasmy	1/115 (0.87%)	0	-	23			PD, early onset	0.446	1	0	0	0
C14347T	V113M	Homoplasmy	1/115 (0.87%)	0	-	21				-	0	1	0	0
T14502C	I58V	Homoplasmy	2/115 (1.74%)	5/672 (0.74%)	2.35	29	Conserved		LHON	0.385	1	1	0	0
14504delA	L57STOP	Homoplasmy	1/115 (0.87%)	0	-	-		Truncated		-	0	0	1	0

Novel mutation

SNP/Mutation	Amino acid substitution	Homoplasmy or heteroplasmy	Frequency T (this study) (%)	Frequency C (mtSNP-Japan)*1 (%)	Frequency (T/C)*2	Grantham value	Evolutionary conservation of the original amino acid	Conformational change	Reported disease association*3	MutPred score*4	No. of cases detected			
											NSCLC		Colon Ca	
											Primary (N=45)	Metastases (N=37)	Primary (N=22)	Metastases (N=11)
**COII**														
G7598A	A5T	Homoplasmy	1/115 (0.87%)	1/672 (0.15%)	5.8	58			Possible LHON helper variant	0.342	0	1	0	0
G7616A	D11N	Homoplasmy	1/115 (0.87%)	0	-	23	Conserved			0.532	0	0	1	0
G7664A	A27T	Homoplasmy	2/115 (1.74%)	6/672 (0.89%)	1.96	58				0.518	0	1	0	1
G7853A	V90I	Homoplasmy	2/115 (1.74%)	30/672 (4.46%)	0.39	29				0.249	2	1	0	0
G7859A	D92N	Homoplasmy	1/115 (0.87%)	2/672 (0.30%)	2.9	23			Progressive Encephalomyopathy	0.226	0	1	1	0
G8075A	A164T	Homoplasmy	1/115 (0.87%)	1/672 (0.15%)	5.8	58				0.74	1	0	0	0
T8223C	L213S	Heteroplasmy or Heterogeneous	1/115 (0.87%)	0	-	145				0.478	0	0	0	1
**ATP6**														
A8531G	N2S	Homoplasmy	1/115 (0.87%)	0	-	46				0.651	1	0	0	0
A8563G	T13A	Homoplasmy	5/115 (4.35%)	44/672 (6.55%)	0.66	53				0.405	2	2	1 w/ liver meta	0
G8584A	A20T	Homoplasmy	5/115 (4.35%)	50/672 (7.44%)	0.58	58				0.553	1	4	0	0
T8602C	F26L	Homoplasmy	1/115 (0.87%)	0	-	22				0.614	1	0	0	0
A8701G	T59A	Homoplasmy	56/114 (49.12%)	425/672 (63.24%)	0.78	58				0.14	29	15	11	6
T8762C	I79T	Homoplasmy	2/115 (0.87%)	2/672 (0.30%)	2.9	89				0.687	1	1	0	0

C8794T	H90Y	Homoplasmy	7/115 (6.09%)	48/672 (7.14%)	0.85	83			Exercise Endurance / Coronary Atherosclerosis risk	0.608	1	3	2 (1 w/ liver meta)	0
A8894T	N123I	Homoplasmy	1/115 (0.87%)	4/672 (0.60%)	1.45	149				0.417	0	1	0	0
G9053A	S176N	Homoplasmy	1/115 (0.87%)	16/672 (2.38%)	0.37	46				0.37	0	0	0	1
C9099A	I191M	Homoplasmy	1/115 (0.87%)	1/672 (0.15%)	5.8	10				0.601	0	1	0	0
T9101G	I192S	Homoplasmy	1/115 (0.87%)	0	-	142			LHOH	0.529	1	0	0	0
**CYTB**														
A14750G	T2A	Homoplasmy	1/115 (0.87%)	4/672 (0.6%)	1.45	58				0.157	1	0	0	0
C14766T	T7I	Homoplasmy	115/115 (100%)	672/672 (100%)	1	89				0.165	45	37	22	11
T14798C	F18L	Homoplasmy	1/115 (0.87%)	0	-	22				0.609	1	0	0	0
G14804A	D20N	Homoplasmy	1/115 (0.87%)	0	-	23				0.716	1	0	0	0
A14870G	I42V	Homoplasmy	1/115 (0.87%)	1/672 (0.15%)	5.8	29				0.326	1	0	0	0
T14934C	F63S	Homoplasmy	1/115 (0.87%)	0	-	155				0.725	0	0	1 w/ liver meta	0
T14979C	I78T	Homoplasmy	5/115 (4.35%)	42/672 (6.25%)	0.7	89				0.239	3	2	1	1
G14985A	R80H	Homoplasmy	1/115 (0.87%)	0	-	29				0.833	1	0	0	0
T15071C	Y109H	Homoplasmy	2/115 (1.74%)	4/672 (0.6%)	2.9	83				0.554	1	1	0	
A15218G	T158A	Homoplasmy	2/115 (1.74%)	10/672 (1.49%)	1.17	58				0.366	1	0	1 w/ liver meta	0
A15236G	I164V	Homoplasmy	3/115 (2.61%)	16/672 (2.38%)	1.1	29				0.224	1	1	0	1
G15254A	V170M	Heteroplasmy or Heterogeneous	2/115 (1.74%)	0	-	21				0.719	0	0	1 w/ liver meta	1
G15314A	A190T	Homoplasmy	1/115 (0.87%)	6/672 (0.89%)	0.98	58				0.501	0	0	1	0
G15323A	A193T	Homoplasmy	1/115 (0.87%)	25/672 (3.72%)	0.23	58				0.517	0	0	1	0
A15326G	T194A	Homoplasmy	115/115 (100%)	672/672 (100%)	1	58				0.452	45	37	22	11
A15363G	N206S	Homoplasmy	1/115 (0.87%)	0	-	46				0.383	0	0	0	1
G15497A	G251S	Homoplasmy	1/115 (0.87%)	25/672 (3.72%)	0.23	56	Conserved		EXIT / Obesity	0.477	0	0	1	0
A15860G	I372V	Homoplasmy	1/115 (0.87%)	22/672 (3.27%)	0.27	29				0.238	0	0	1	0

Somatic mutation — Novel mutations found in this study are shown in red.

*1 The SNP frequency in persons with Parkinson's disease, Alzheimer's disease and type 2 diabetes w/ and w/o vascular lesion, men w/ and w/o juvenile obesity, and centenarians of Gifu and Tokyo in the database of GiiB-JST mtSN, the human mitochondrial genome SNP database (http://mtsnp.

*2 Calculated on the basis of the SNP frequency (C) in the database of GiiB-JST mtSN and the SNP frequency (T) in this study.

*3 Data from MITOMAP database (http://www.mitomap.org/MITOMAP). PD; Parkinson's disease, AD; Alzheimer's disease, AMS; 4-Acetamido-4′maleimidyldystilbene-2,2′-disulphonic acid, DEAF; Maternally inherited DEAFness or aminoglycoside-induced DEAFness, DM: Type 2 diabetes mellitu, DMDF; Diabetes Mellitus + DeaFness, EXIT; Exercise int0lerance. HCM; Hypertrophic cardiomyopathy, LHON; Leber hereditary optic neuropathy, LDYT; Leber's hereditary optic neuropathy and DYsTonia, MELAS: Mitochondrial Encephalomyopathy, Lactic Acidosis, and Stroke-like episodes, MS; Multiple sclerosis, NIDDM: Non-insulin-dependent diabetes mellitus,

*4 Predicted pathogenicity score (Ref. 18). The amino acid variants showing mor ethan 0.7 are considered as a high pathogenicity score group.

Supplementary Table S3. Synonymous SNPs and SNVs in the *ND* genes in NSCLC and colon cancer clinical samples.

NSCLC primary tissue (45 cases)

None (grey) | Novel mutation (red)

ID	Age/Sex	Histology	TNM*1	ND1	ND2	ND3	ND4L	ND4	ND5	ND6	COII	ATP6	CYTB
LgCa-150	71/M	Squamous cell carcinoma	T3N2M0		A4769G (M100M)			G10801A (L14L)<br>C11536T (Y259Y)<br>G11719A (G320G)	C12705T (I123I)				C14944T (I66I)
LgCa-151	57/M	Squamous cell carcinoma	T2N0M0		A4769G (M100M)<br>C4883T (P138P)	C10400T (T114T)		T10873C (P38P)<br>G11719A (G320G)	C12705T (I123I)<br>T13656C (L440L)	C14668T (M2M)			T14783C (L13L)<br>G15043A (G99G)<br>T15139C (Y131Y)<br>G15301A (L185L)
LgCa-152	59/F	Adenocarcinoma	T4N0M0		A4769G (M100M)<br>C4883T (P138P)<br>G5261A (W264W)	C10400T (T114T)		T10873C (P38P)<br>G11719A (G320G)	C12705T (I123I)	C14668T (M2M)			T14783C (L13L)<br>G15301A (L185L)<br>G15314A (L185L)
LgCa-153	64/M	Large cell carcinoma	T4N2M1		A4769G (M100M)<br>A4958G (M163M)	C10400T (T114T)		T10873C (P38P)<br>T11017C (S86S)<br>G11719A (G320G)	C12705T (I123I)<br>G12771A (E145E)<br>A13563G (L409L)	G14364A (L104L)			T14783C (L13L)<br>G15043A (G99G)<br>G15301A (L185L)
LgCa-154	76/F	Squamous cell carcinoma	T1N0M0		A4769G (M100M)<br>C4883T (P138P)	C10400T (T114T)		T10873C (P38P)<br>G11719A (G320G)<br>C12088T (P443P)	G12651C (L105L)<br>C12705T (I123I)	C14668T (M2M)			T14783C (L13L)<br>G15043A (G99G)<br>G15301A (L185L)
LgCa-157	63/M	Squamous cell carcinoma	T1N0M0		A4769G (M100M)<br>C4883T (P138P)<br>G5417A (Q316Q)	C10400T (T114T)		T10873C (P38P)<br>T11215T (Y152Y)<br>G11719A (G320G)	C12705T (I123I)	C14668T (M2M)			T14783C (L13L)<br>G15043A (G99G)<br>G15301A (L185L)
LgCa-159	59/M	Squamous cell carcinoma	T2N1M0		C4505T (T12T)<br>A4769G (M100M)<br>T5108C (T213T)			T10873C (P38P)<br>G11719A (G320G)	C12705T (I123I)<br>A13563G (L409L)	T14200C (W158W)<br>C14281T (G131G)<br>G14569A (S35S)	G7600A (A5A)	T9165C (V213V)	T14783C (L13L)<br>G15043A (G99G)<br>G15301A (L185L)
LgCa-161	71/M	Adenocarcinoma	T2N1M0		A4769G (M100M)<br>A4958G (M163M)			T10873C (P38P)<br>T11017C (S86S)<br>G11719A (G320G)	C12705T (I123I)<br>G12771A (E145E)	G14364A (L104L)			T14783C (L13L)<br>G15043A (G99G)<br>G15301A (L185L)
LgCa-162	62/M	Large cell carcinoma	T1N0M0	C4071T (Y255Y)<br>A4164G (M286M)	A4769G (M100M)<br>A5351G(L294L)			T10873C (P38P)<br>G11719A (G320G)	C12405T (L23L)<br>C12705T (I123I)		T7684C (L33L)		T14783C (L13L)<br>G15043A (G99G)<br>G15301A (L185L)
LgCa-163	57/M	Adenocarcinoma	T1N0M0		A4769G (M100M)<br>C4883T (P138P)<br>T5291C (N274N)			T10873C (P38P)<br>G11719A (G320G)	C12705T (I123I)	T14386C (L96L)<br>C14668T (M2M)	G8020A (P145P)	C8964T (T146T)	T14783C (L13L)<br>G15043A (G99G)<br>G15301A (L185L)
LgCa-164	37/M	Adenocarcinoma	T1N0M0		A4769G (M100M)<br>A4958G (M163M)			T10873C (P38P)<br>T11017C (S86S)<br>G11719A (G320G)	C12705T (I123I)<br>G12771A (E145E)	G14364A (L104L)			T14783C (L13L)<br>G15043A (G99G)<br>G15301A (L185L)
LgCa-165	79/F	Adenocarcinoma	T2N2M0	G3531A (P75P)	A4769G (M100M)<br>C4883T (P138P)			T10873C (P38P)<br>T11260C (T167T)<br>G11719A (G320G)	C12705T (I123I)<br>T12957C (N207N)	C14668T (M2M)			T14783C (L13L)<br>G15043A (G99G)<br>G15301A (L185L)
LgCa-166	71/M	Squamous cell crcinomaa	T4N2M0		A4769G (M100M)			G11719A (G320G)<br>G12007A (W416W)	G12372A (L12L)<br>C12705T (I123I)				
LgCa-167	56/F	Adenocarcinoma	T1N0M0		A4769G (M100M)<br>G5231A (L254L)<br>G5417A (Q316Q)			G11719A (G320G)	G12372A (L12L)<br>C12705T (I123I)	A14464G (T70T)		T8598C (I24I)	T15067C (F107F)<br>G15043A (G99G)<br>G15106A (L120L)
LgCa-168	61/F	Adenocarcinoma	T1N0M0	C4140T (P278P)	A4769G (M100M)	C10400T (T114T)	G10646A (V59V)	T10873C (P38P)<br>G11719A (G320G)	C12549T (T71T)<br>C12705T (I123I)<br>A13152G (L272L)			A8700G (M58M)<br>T8793C (P89P)<br>G8856A (A110A)	T14783C (L13L)<br>C15040T (I98I)<br>G15043A (G99G)<br>G15301A (L185L)
LgCa-169	72/M	Squamouse cell carcinoma	T1N1M0		A4769G (M100M)<br>C4883T (P138P)	C10400T (T114T)		T10873C (P38P)<br>G11719A (G320G)	C12705T (I123I)	T14374C (V100V)<br>C14668T (M2M)		A8706G (M60M)	T14783C (L13L)<br>G15043A (G99G)<br>G15301A (L185L)
LgCa-173	74/M	Squamouse cell carcinoma	T3N0M0	C4071T (Y255Y)<br>A4164G (M286M)	A4769G (M100M)<br>A5351G(L294L)			T10873C (P38P)<br>G11719A (G320G)	C12405T (L23L)<br>C12705T (I123I)		T7684C (L33L)		T14783C (L13L)<br>G15043A (G99G)<br>G15301A (L185L)
LgCa-174	59/M	Adenocarcinoma	T2N1M0	C3921T (S205S)<br>T4248C (I314I)	A4769G (M100M)			A11023G (P88P)<br>C11536T (Y259Y)<br>C11647T (L296L)<br>G11719A (G320G)	C12705T (I123I)				
LgCa-175	46/F	Adenocarcinoma	T1N0M0		A4769G (M100M)<br>G4820A (E117E)<br>T5021C (I184I)			A11239G (L160L)<br>G11719A (G320G)	G13590A (L418L)	A14587G (G29G)			

LgCa-176	80/M	Squamouse cell carcinoma	T1N0M0	C3970T (L222L)	C5049T (L194L)<br>G5147A (T226T)	G10310A (L84L)		C10976T (L73L)<br>G11719A (G320G)	C12633T (S99S)<br>C12882T (F182F)	G14476A (V66V)			
LgCa-177	43/F	Squamouse cell carcinoma	T1N0M0		A4769G (M100M)<br>C4883T (P138P)	C10400T (T114T)		C11215T (Y152Y)<br>G11719A (G320G)	C12705T (I123I)	C14668T (M2M)			T14783C (L13L)<br>G15043A (G99G)<br>G15301A (L185L)
LgCa-178	58/M	Squamouse cell carcinoma	T1N1M0	C3546T (T80T)<br>A4200T (L298L)	A4769G (M100M)<br>C4883T (P138P)	C10400T (T114T)		T10873C (P38P)<br>G11719A (G320G)<br>G11719A (G320G)	C12705T (I123I)				T14783C (L13L)<br>G15043A (G99G)<br>G15301A (L185L)
LgCa-179	54/F	Adenocarcinoma	T1N0M0		A4769G (M100M)<br>C4883T (P138P)	C10400T (T114T)		T10873C (P38P)<br>C11215T (Y152Y)	C12705T (I123I)	C14668T (M2M)			T14783C (L13L)<br>G15043A (G99G)<br>G15301A (L185L)
LgCa-180	68/M	Adenocarcinoma	T2N1M0	T4248C (I314I)	G4655A (T62T)<br>A4769G (M100M)			G10801A (L14L)<br>C11536T (Y259Y)<br>C11647T (L296L)<br>G11719A (G320G)	C12705T (I123I)				C14944T (I66I)
LgCa-181	64/M	Squamouse cell carcinoma	T1N2M0		A4715G (G82G)<br>A4769G (M100M)	C10400T (T114T)		T10873C (P38P)<br>C11215T (Y152Y)<br>G11719A (G320G)	C12705T (I123I)	C14668T (M2M)	A8188G (201G)	T9090C (S188S)	T14783C (L13L)<br>G15043A (G99G)<br>T15184C (I146I)<br>G15301A (L185L)
LgCa-182	46/M	Adenocarcinoma	T1N2M0		A4769G (M100M)<br>A4958G (M163M)	C10400T (T114T)		T10873C (P38P)<br>T11017C (S86S)<br>G11719A (G320G)	C12705T (I123I)<br>G12771A (E145E)	G14364A (L104L)			T14783C (L13L)<br>G15043A (G99G)<br>G15301A (L185L)
lgCa-183	67/M	Squamouse cell carcinoma	T2N0M0	T4117C (L271L)	A4769G (M100M)<br>G4820A (E117E)			G11719A (G320G)	G13590A (L418L)		G8206A (M207M)		
LgCa-184	52/M	Pleomorphic carcinoma	T3N0M0		A4769G (M100M)<br>G5231A (L254L)<br>G5237A (P256P)<br>G5417A (Q316Q)			G11719A (G320G)	G12372A (L12L)<br>C12705T (I123I)				T15067C (F107F)
LgCa-187	73/M	Adenocarcinoma	T1N0M0	C3970T (L222L)	A4769G (M100M)<br>C5049T (L194L)<br>G5147A (T226T)	G10310A (L84L)		C10976T (L73L)<br>G11719A (G320G)	C12633T (S99S)<br>C12882T (F182F)	G14476A (V66V)			
LgCa-188	59/M	Squamouse cell carcinoma	T1N0M0		A4769G (M100M)<br>C4883T (P138P)	C10400T (T114T)		T10873C (P38P)<br>G11719A (G320G)	C12705T (I123I)	C14668T (M2M)		T8614C (L30L)<br>A8697G (M57M)	T14783C (L13L)<br>G15043A (G99G)<br>G15301A (L185L)
LgCa-189	73/M	Squamouse cell carcinoma	T1N1M0	A3759G (L151L)	A4769G (M100M)<br>C4883T (P138P)<br>A5153G (L228L)	C10400T (T114T)		T10873C P83P)<br>T11569C (I270I)<br>G11719A (G320G)	C12705T (I123I)	T14319C (N119N)		A9180G (V218V)	T14783C (L13L)<br>G15043A (G99G)<br>G15301A (L185L)
LgCa-190	62/M	Adenocarcinoma	T2N1M0		A4769G (M100M)<br>G5147A (T226T)<br>G5417A (Q316Q)	T10235C (A59A)		T10882C (F41F)<br>G11719A (G320G)	C12705T (I123I)	A14233G (D147D)			A14893G (L49L)
LgCa-191	50/M	Adenocarcinoma	T2N2M0	C3846T (P180P)	A4769G (M100M)		C10607T (L46L)	G11719A (G320G)				G8790A (88L)	G15043A (G99G)
LgCa-192	54/F	Adenocarcinoma	T4N0M0	C3921T (S205S)<br>T4248C (I314I)	A4769G (M100M)			C11536T (Y259Y)<br>C11647T (L296L)<br>G11719A (G320G)	C12705T (I123I)				G15172A (G142G)
LgCa-193	76/M	Squamouse cell carcinoma	T1N0M0		A4769G (M100M)<br>C4883T (P138P)			T10873C (P38P)<br>C11215T (Y152Y)<br>G11719A (G320G)	C12705T (I123I)	C14668T (M2M)			T14783C (L13L)<br>G15043A (G99G)<br>G15301A (L185L)
LgCa-194	41/M	Adenocarcinoma	T2N2M0		A4769G (M100M)<br>C4883T (P138P)<br>A5153G (L228L)	G10397A (W113W)<br>C10400T (T114T)		T10873C (P38P)<br>G11719A (G320G)	C12705T (I123I)<br>C13029T (P231P)<br>T13437C (P367P)			A9180G (V218V)	T14783C (L13L)<br>G15043A (G99G)<br>G15301A (L185L)
LgCa-195	66/M	Bronchioalveolar carcinoma	T1N0M0		A4769G (M100M)<br>C4883T (P138P)	C10400T (T114T)		T10873C (P38P)<br>C11215T (Y152Y)<br>G11719A (G320G)	C12705T (I123I)	C14668T (M2M)			T14783C (L13L)<br>G15043A (G99G)<br>G15301A (L185L)
LgCa-196	64/M	Adenocarcinoma	T1N0M0		C4625T (S52S)<br>A4769G (M100M)			G11719A (G320G)	C12853T (L173L)<br>T13437C (P367P)				G15346A (L200L)
LgCa-197	59/M	Bronchioalveolar carcinoma	T1N0M0		A4769G (M100M)	C10400T (T114T)		T10873C (P38P)<br>G11719A (G320G)	C12705T (I123I)	T14308C (G122G)			T14783C (L13L)<br>G15043A (G99G)<br>G15301A (L185L)
LgCa-198	35/M	Squamous cell carcinoma	T?N1M0		A4769G (M100M)<br>A4958G (M163M)	C10400T (T114T)		T10873C (P38P)<br>T11017C (S86S))<br>G11719A (G320G)<br>T11722C (L321L)	C12705T (I123I)<br>G12771A (E145E)	G14364A (L104L)			T14783C (L13L)<br>G15043A (G99G)<br>G15301A (L185L)
LgCa-199	63/M	Adenocarcinoma	T1N1M0		A4769G (M100M)<br>G5231A (L254L)<br>G5417A (Q316Q)			G11719A (G320G)<br>A11731G (S324S)	G12372A (L12L)<br>C12705T (I123I)				T14968C (N74N)<br>T15067C (F107F)

LgCa-200	58/F	Adenocarcinoma	T1N0M0	C4071T (Y255Y)<br>A4164G (M286M)	A4769G (M100M)<br>A5351G(L294L)	C10400T (T114T)		T10873C (P38P)<br>G11719A (G320G)	C12405T (L23L)<br>C12705T (I123I)		T7684C (L33L)		T14783C (L13L)<br>G15043A (G99G)<br>G15301A (L185L)
LgCa-201	64/M	Adenocarcinoma	T1N0M0		A4769G (M100M)<br>A4895G (M163M)			T11437C (A226A)<br>G11719A (G320G)				C8829T (N101N)	C15223T (D159D)
LgCa-202	76/M	Adenocarcinoma	T1N0M0		A4769G (M100M)<br>T4859C (L130L)<br>C4883T (P138P)	C10400T (T114T)		T10873C (P38P)<br>G11719A (G320G)	C12705T (I123I)	T14635C (V13V)<br>C14668T (M2M)			T14783C (L13L)<br>G15043A (G99G)<br>G15301A (L185L)
LgCa-205	74/F	Squamous cell carcinoma	T1N1M0		A4769G (M100M)<br>C4883T (P138P)	C10400T (T114T)		T10873C (P38P)<br>G11719A (G320G)	C12705T (I123I)<br>T13656C (L440L)	C14668T (M2M)			T14783C (L13L)<br>G15043A (G99G)<br>T15139C (Y131Y)<br>G15301A (L185L)

*1 TNM (Tumour/Node/Metastasis) clasification for staging of cancer.

NSCLC metastases (37 cases)

None — Novel mutation

Sample #		Histology	Metastatic site	ND1	ND2	ND3	ND4L	ND4	ND5	ND6	COII	ATP6	CYTB
LuAdBrM-1	-	Adenocarcinoma	Brain		A4769G (M100M)<br>A4958G (M163M)	C10400T (T114T)	G10646A (V59V)	T10873C (P38P)<br>T11017C (S86S)<br>G11719A (G320G)	C12705T (I123I)<br>G12771A (E145E)	G14364A (L104L)			T14783C (L13L)<br>G15043A (G99G)<br>G15301A (L185L)
LuAdBrM-2	64/M	Adenocarcinoma	Brain		A4769G (M100M)	G10310A (L84L)		G11719A (G320G)	A14133G (L599L)			C8844T (I106I)	G15346A (L200L)
LuAdBrM-3	–	Adenocarcinoma	Brain		A4769G (M100M)<br>A4958G (M163M)<br>G4991A (Q174Q)	C10400T (T114T)		T10873C (P38P)<br>T11017C (S86S)<br>G11719A (G320G)<br>T11722C (L321L)	C12705T (I123I)<br>G12771A (E145E)	G14364A (L104L)			T14783C (L13L)<br>G15301A (L185L)
LuAdBrM-4	–	Adenocarcinoma	Brain		G4655A (T62T)<br>A4769G (M100M)			C11536T (Y259Y)<br>C11647T (L296L)<br>G11719A (G320G)	C12705T (I123I)				
LuAdBrM-5	79/M	Adenocarcinoma	Brain		A4769G (M100M)	C10400T (T114T)		T10873C (P38P)<br>G11719A (G320G)	C12705T (I123I)<br>T13015C (L227L)	T14308C (G122G)			T14783C (L13L)<br>G15301A (L185L)
LuAdBrM-6	–	Adenocarcinoma	Brain	G3483A (E59E)	G5231A (L254L)<br>A4769G (M100M)<br>G5417A (Q316Q)			G11719A (G320G)	G12372A (L12L)<br>C12705T (I123I)<br>T13602C (Y422Y)				T15067C (F107F)<br>G15301A (L185L)
LuAdBrM-7	49/M	Adenocarcinoma	Brain		A4769G (M100M)	A10403G (E115E)		G11719A (G320G)					
LuAdBrM-8	70/M	Adenocarcinoma	Brain		A4769G (M100M)	C10400T (T114T)		T10873C (P38P)<br>G11719A (G320G)	C12705T (I123I)	T14308C (G122G)			T14783C (L13L)<br>G15043A (G99G)<br>G15301A (L185L)
LuAdBrM-9	–	Adenocarcinoma	Brain		A4769G (M100M)<br>G5147A (T226T)	G10310A (L84L)		G11719A (G320G)	T13962C (L542L)<br>A14133G (L599L)			G8856A (A110A)	G15346A (L200L)
LuAdBrM-10	51/M	Adenocarcinoma	Brain		A4769G (M100M)<br>C4883T (P138P)	C10400T (T114T)		T10873C (P38P)<br>T11017C (S86S)<br>G11719A (G320G)	C12705T (I123I)	C14668T (M2M)			T14783C (L13L)<br>G15043A (G99G)<br>G15301A (L185L)
LuAdBrM-11	–	Adenocarcinoma	Brain	G4140T (P278P)	A4769G (M100M)	C10400T (T114T)		T10873C (P38P)<br>G11719A (G320G)	C12549 T (T71T)<br>C12705T (I123I)<br>A13152G (L272L)		C7948T (Y121Y)	G8793C (P89P)<br>G8856A (A110A)	T14783C (L13L)<br>G15043A (G99G)<br>G15301A (L185L)
LuAdBrM-12	–	Adenocarcinoma	Brain		A4769G (M100M)<br>A4859G (L130L)			T11437C (A226A)<br>G11719A (G320G)	T13641C (P435P)<br>A13887G (L517L)			C8829T (N101N)	C15223T (D159D)
LuAdBrM-13	46/M	Adenocarcinoma	Brain		A4769G (M100M)<br>C4883T (P138P)	C10400T (T114T)		T10873C (P38P)<br>G11719A (G320G)	C12705T (I123I)<br>A13104G (G256G)<br>T13641C (P435P)<br>A13887G (L517L)	C14668T (M2M)			T14783C (L13L)<br>G15043A (G99G)<br>G15301A (L185L)
LuAdBrM-14	–	Adenocarcinoma	Brain		A4769G (M100M)<br>G5231A (L254L)<br>G5417A (Q316Q)			G11719A (G320G)	G12372A (L12L)<br>G12651A (L105L)<br>C12705T (I123I)	C14668T (M2M)			T15067C (F107F)
LuAdBrM-15	50/M	Adenocarcinoma	Brain		A4769G (M100M)<br>A4958G (M163M)	C10400T (T114T)		T10873C (P38P)<br>T11017C (S86S)<br>G11719A (G320G)<br>T11944C (L395L)	C12705T (I123I)<br>G12771A (E145E)		G7852A (E89E)		T14783C (L13L)<br>G15043A (G99G)<br>G15301A (L185L)
LuAdBrM-16	69/M	Adenocarcinoma	Brain		A4769G (M100M)<br>C4883T (P138P)	C10400T (T114T)		T10873C (P38P)<br>G11719A (G320G)	C12705T (I123I)<br>A13104G (G256G)	G14364A (L104L)			T14783C (L13L)<br>G15043A (G99G)<br>G15301A (L185L)
LuAdBrM-17	50/F	Adenocarcinoma	Brain		A4769G (M100M)<br>A4895G (M142M)			T11437C (A226A)<br>G11719A (G320G)				C8829T (N101N)	C15223T (D159D)

LuAdBrM-18	–	Adenocarcinoma	Brain	T4248C (I314I)	G4655A (T62T)<br>A4769G (M100M)			C11536T (Y259Y)<br>C11647T (L296L)<br>G11719A (G320G)	C12705T (I123I)				
LuAdBrM-19	47/M	Adenocarcinoma	Brain		A4769G (M100M)<br>A4895G (M142M)			G11719A (G320G)				C8829T (N101N)	C15223T (D159D)
LuAdBrM-20	70/M	Adenocarcinoma	Brain	C4071T (Y255Y)<br>A4164G (M286M)	A4769G (M100M)<br>A5351G (L924L)	C10400T (T114T)		T10873C (P38P)<br>G11719A (G320G)	C12405T (L23L)<br>C12705T (I123I)		T7684C (L33L)		T14783C (L13L)<br>G15043A (G99G)<br>G15301A (L185L)
LuAdBrM-22	72/M	Adenocarcinoma	Brain		A4769G (M100M)<br>C4883T (P138P)	C10400T (T114T)		T10873C (P38P)<br>G11719A (G320G)	C12705T (I123I)	A14605G (P23P)<br>C14668T (M2M)	G8020A (P145P)	C8964T (T146T)	T14783C (L13L)<br>G15043A (G99G)<br>G15301A (L185L)
LuAdBrM-23	–	Adenocarcinoma	Brain		A4769G (M100M)<br>C4883T (P138P)	C10400T (T114T)		C12088T (P443P)<br>G11719A (G320G)	G12651C (L105L)<br>C12705T (I123I)	C14668T (M2M)			T14783C (L13L)<br>G15043A (G99G)<br>G15301A (L185L)
LuAdBrM-24	68/F	Adenocarcinoma	Brain		A4769G (M100M)<br>A4958G (M163M)	C10400T (T114T)		T10873C (P38P)<br>T11017C (S86S)	C12705T (I123I)<br>G12771A (E145E)	G14364A (L104L)	T7741C (N52N)		T14783C (L13L)<br>G15043A (G99G)<br>G15301A (L185L)
LuAdBrM-25	74/F	Adenocarcinoma	Brain	C3970T (L222L)	A4769G (M100M)<br>C5049T (L194L)<br>G5147A (T262T)	G10310A (L84L)		C10976T (L73L)<br>G11719A (G320G)	C12633T (S99S)<br>C12882T (F182F)	G14476A (V66V)			
LuPoBrM-1	–	Adenocarcinoma	Brain		C4625T (S52S)<br>A4769G (M100M)<br>T4856C (L129L)			G11719A (G320G)	T12714C (I126I)<br>C12853T (L173L)<br>T13437C (P367P)				G15346A (L200L)
LuPoBrM-2	–	Adenoarcinoma	Brain		T4742C (N91N)<br>A4769G (M100M)			G11719A (G320G)			C7909T (Y108Y)<br>T8167A (G194G)		G15148A (P134P)<br>G15346A (L200L)
LuPoBrM-3	–	Adenoarcinoma	Brain	T4248C (I314I)	T4742C (N91N)<br>A4769G (M100M)			G11719A (G320G)	C12705T (I123I)				
LuSQBrM-1	–	Squamous carcinoma	Brain	G3882A (E192E)<br>C4071T (Y255Y)	A4769G (M100M)<br>C4850T (G127G)			T10873C (P38P)<br>C11389T (Y210Y)<br>C11665T (L302L)<br>G11719A (G320G)<br>G11914A (T385T)<br>T12091T (I444I)	C12705T (I123I)		A7960G (P125P)		T14783C (L13L)<br>G15043A (G99G)<br>G15301A (L185L)
LuSQBrM-2	62/F	Squamous carcinoma	Brain		A4769G (M100M)<br>C4883T (P138P)	C10400T (T114T)		T10873C (P38P)<br>G11719A (G320G)	C12705T (I123I)	C14668T (M2M)		T8614C (L30L)	T14783C (L13L)<br>G15043A (G99G)<br>G15301A (L185L)
LuSQBrM-3	63/M	Squamous carcinoma	Brain		A4769G (M100M)<br>C4883T (P138P)	C10400T (T114T)<br>T10238C (I60I)		T10873C (P38P)<br>G11719A (G320G)	C12705T (I123I)	A14605G (P23P)<br>C14668T (M2M)	G8020A (P145P)	C8964T (T146T)	T14783C (L13L)<br>G15043A (G99G)<br>G15301A (L185L)
LuSQBrM-4	65/M	Squamous carcinoma	Brain		A4769G (M100M)<br>C4883T (P138P)	C10400T (T114T)		T10873C (P38P)<br>G11719A (G320G)	C12705T (I123I)	C14668T (M2M)			T14783C (L13L)<br>G15043A (G99G)<br>G15301A (L185L)
LuSQBrM-5	79/M	Squamous carcinoma	Brain	C3970T (L222L)	A4769G (M100M)<br>C5049T (L194L)<br>G5147A (T62T)	C10400T (T114T)		T10873C (P38P)<br>G11719A (G320G)	C12633T (S99S)<br>C12882T (F182F)	G14476A (V66V)			
LuSQBrM-6	–	Squamous carcinoma	Brain	T4117C (L271L)	A4769G (M100M)<br>G4820A (G117G)			G11719A (G320G)	G13590A (L418L)		G8206A (M207M)		
LuSQBrM-7	–	Squamous carcinoma	Brain		A4769G (M100M)<br>G5147A (T262T)	G10310A (L84L)		T10873C (P38P)<br>G11719A (G320G)	T13962C (L542L)<br>A14133G (L599L)			G8856A (A110A)	G15346A (L200L)
LuSQBrM-8	–	Squamous carcinoma	Brain	T3552A (A82A)<br>T3826C (L174L)	A4715G (G82G)<br>A4769G (M100M)	C10400T (T114T)		G11914A (T385T)<br>G11719A (G320G)	C12705T (I123I)<br>A13263G (Q309Q)				T14783C (L13L)<br>G15043A (G99G)<br>G15301A (L185L)
LgCa-194	41/M	Adenocarcinoma	Brain		A4769G (M100M)<br>A5153G (L228L)<br>C4883T (P138P)			G11719A (G320G)					
LuAdBrM-26	54/F	Adenocarcinoma	Brain	C3970T (L222L)		G10310A (L84L)		G11719A (G320G)	C12882T (F182F)		G7702A (L39L)<br>T7738C (T51T)		

Colon cancer primary tissue (22 cases)

None | Novel mutation

Case #	Age/Sex	Histology	TNM*1	ND1	ND2	ND3	ND4L	ND4	ND5	ND6		COII	ATP6	CYTB
ColoCa-1	69/M	Adenocarcinoma	T3N1M1	T4248C (I314I)	G4655A (T62T)<br>A4769G (M100M)	G10325A (M89M)		T10873C (P38P)<br>C11536T Y259Y)<br>C11647T (L296L)<br>G11719A (G320G)	C12705T (I123I)					
ColoCa-2	65/M	Adenocarcinoma	T3N2M0		A4769G (M100M)<br>A4793G (M108M)<br>T5108C (T213T)	C10400T (T114T)		T10873C (P38P)<br>G11719A (G320G)<br>G11914A (T385T)	C12705T (I123I)	G14569A (S35S)		C7867T (S94S)<br>T8200C (S205S)		T14783C (L13L)<br>G15043A (G99G)<br>G15301A (L185L)
ColoCa-3	71/M	Adenocarcinoma	T3N1M0		A4769G (M100M)<br>C4883T (P138P)	A10397G (W113W)<br>C10400T (T114T)		T10873C (P38P)<br>G11719A (G320G)<br>A11746G (L329L)<br>T11944C (L395L)	C12705T (I123I)<br>A13278G (M315M)				A9180G (V218V)	T14783C (L13L)<br>G15043A (G99G)<br>G15301A (L185L)
ColoCa-4	50/M	Adenocarcinoma	T4N4M0	C3970T (L222L)	A4769G (M100M)<br>C5049T (L194L)<br>G5147A (Q316Q)	G10310A (L84L)		C10976T (L73L)<br>G11719A (G320G)	C12633T (S99S)<br>C12882T (F182F)	G14476A (V66V)				
ColoCa-5	67/M	Adenocarcinoma	T4N4M0		A4769G (M100M)<br>G5231A (L254L)<br>G5237A (P256P)<br>G5417A (Q316Q)		T10724C (Y85Y)	G11719A (G320G)	G12372A (L12L)<br>C12705T (I123I)					T15067C (F107F)
ColoCa-6	67/F	Adenocarcinoma	P(T?N0M1)<br>(Liver meta)		A4769G (M100M)<br>C4883T (P138P)	C10400T (T114T)		T10873C (P38P)<br>C11215T (Y152Y)<br>G11719A (G320G)		C14668T (M2M)		T7783C (L67L)		T14783C (L13L)<br>G15043A (G99G)<br>G15301A (L185L)<br>A15874G (M376M)
ColoCa-7	52/F	Adenocarcinoma	T4N2M1<br>(Liver meta)		A4769G (M100M)<br>C4883T (P138P)			T10873C (P38P)<br>G11719A (G320G)	C12705T (I123I)<br>A13104G (G256G)<br>T13641C (P435P)<br>A13887G (L517L)	C14668T (M2M)				T14783C (L13L)<br>G15043A (G99G)<br>G15301A (L185L)<br>C15518T (L258L)
ColoCa-8	61/M	Adenocarcinoma	T3N0M0	C3408T (R34R)<br>T4248C (I314I)	A4769G (M100M)			G11719A (G320G)	C12705T (I123I)<br>C14067T (T577T)					
ColoCa-9	66/M	Adenocarcinoma	T2N0M0	G4092A (K262K)	A4769G (M100M)			T10873C (P38P)<br>G11719A (G320G)	C12705T (I123I)			C7747T (I55I)	A9180G (V218V)	T14783C (L13L)<br>G15043A (G99G)<br>G15301A (L185L)<br>A15724G (W326W)
ColoCa-10	64/M	Adenocarcinoma	T3N0M0		A4769G (M100M)<br>C4883T (P138P)<br>A5153G (L228L)			T10873C (P38P)<br>C11422T (V221V)<br>G11719A (G320G)	C12705T (I123I)<br>A14013G (E559E)	C14668T (M2M)				T14783C (L13L)<br>G15043A (G99G)<br>G15301A (L185L)
ColoCa-11	61/F	Adenocarcinoma	T3N1M0		A4769G (M100M)<br>C4883T (P138P)			T10873C (P38P)<br>C11215T (Y152Y)<br>G11719A (G320G)		C14668T (M2M)				T14783C (L13L)<br>G15043A (G99G)<br>G15301A (L185L)<br>A15874G (M376M)
ColoCa-12	53/F	Adenocarcinoma	T3N1M0	G3882A (E192E)<br>C4071T (Y255Y)	A4769G (M100M)<br>C4850T (G127G)			T10873C (P38P)<br>G11719A (G320G)	C12705T (I123I)					T14783C (L13L)<br>G15043A (G99G)<br>G15301A (L185L)
ColoCa-13	66/M	Adenocarcinoma	T3N1M1<br>(Liver meta)	G3970T (L222L)	A4769G (M100M)<br>C5049T (L194L)<br>G5147A (T226T)	G10310A (L84L)		C10976T (L73L)<br>G11719A (G320G)	C12633T (S99S)<br>C12882T (F182F)	C14476A (V66V)	Matched case 1			
ColoCa-14	74/F	Adenocarcinoma	T1N0M0	C3970T (L222L)	A4769G (M100M)<br>G4820A (E117E)			G11719A (G320G)		C14476A (V66V)				
ColoCa-15	82/F	Adenocarcinoma	T1N0M0	C3495T (P63P)<br>C4071T (Y255Y)<br>A4164G (M286M)	A4769G (M100M)<br>T5021C(I184I)<br>A5351G(L294L)	C10400T (T114T)		T10873C (P38P)<br>G11719A (G320G)	C12405T (L23L)<br>C12705T (I123I)<br>G13707A (L457L)<br>C13782T (I482I)			T7684C (L33L)		T14783C (L13L)<br>A15043G (G99G)<br>A15301G (L185L)
ColoCa-16	66/M	Carcinoid	T1N1M0		A4769G (M100M)<br>C4883T (P138P)	C10400T (T114T)		T10873C (P38P)<br>G11719A (G320G)	C12705T (I123I)	C14668T (M2M)		G8020A (P145P)	C8964T (T146T)	T14783C (L13L)<br>G15043A (G99G)<br>G15301A (L185L)
ColoCa-17	75/F	Adenocarcinoma	T1N0M0	C3970T (L222L)	A4769G (M100M)		T10535C (Y22Y)<br>G10586A (S39S)	T10790C (L11L)<br>G11719A (G320G)				A7828G (L81L)		
ColoCa-18	54/F	Adenocarcinoma	T4N1M1<br>(Liver meta)		A4769G (M100M)<br>A5498G (M163M)	C10400T (T114T)		T10873C (P38P)<br>T11017C (S86S)<br>G11719A (G320G)	C12705T (I123I)<br>G12771A (E145E)	G14364A (L104L)<br>T14470C (G68G)				T14783C (L13L)<br>G15043A (G99G)<br>G15301A (L185L)
ColoCa-19	56/M	Adenocarcinoma	T3N0M0	G3531A (P75P)	A4769G (M100M)<br>C4883T (P138P)	C10400T (T114T)		T10873C (P38P)<br>G11719A (G320G)	C12705T (I123I)<br>T12957C (N207N)	C14668T (M2M)				T14783C (L13L)<br>G15043A (G99G)<br>G15301A (L185L)

ColoCa-20	62/M	Adenocarcinoma	T1N0M0		A4769G (M100M)<br>G4820A (E117E)<br>G5231A (L254L)<br>G5417A (Q316Q)			G11719A (G320G)	C12705T (I123I)<br>G12372A (L12L)					
ColoCa-21	63/M	Adenocarcinoma	T?N?M1<br>(Liver meta)		A4769G (M100M)<br>A4958G (M163M)	C10400T (T114T)		T10873C (P38P)<br>T11017C (S86S)<br>G11719A (G320G)	C12705T (I123I)<br>G12771A (E145E)	G14364A (L104L)				T14783C (L13L)<br>G15043A (G99G)<br>G15301A (L185L)
ColoCa-22	67/M	Adenocarcinoma	T4N1M1<br>(Liver meta)		A4769G (M100M)<br>A5084G (L205L)	C10400T (T114T)		G11719A (G320G)<br>G11914A (T385T)			Matched case 2		A9120G (L198L)	C15535T (N263N)

*1 TNM (Tumour/Node/Metastasis) clasification for staging of cancer.

Colon cancer metastases (11 cases) — None (grey) — Novel mutation (red)

Cae #	Age/Sex	Histology	Metastasis site	ND1	ND2	ND3	ND4L	ND4	ND5	ND6		COII	ATP6	CYTB
CoAdBrM-1	–	Adenocarcinoma	Brain			C10400T (T114T)		G11719A (G320G)	C12882T (F182F)					
ColonLivM-1	–	-	Liver		A4958G (M163M)	C10400T (T114T)		T10873C (P38P)<br>T11017C (S86S)<br>G11719A (G320G)		G14364A (L104L)				T14783C (L13L)<br>G15043A (G99G)<br>G15301A (L185L)
ColonLivM-2	–	-	Liver		C4883T (P138P)	C10104T (L16L)<br>C10400T (T114T)		T10873C (P38P)<br>G11719A (G320G)	C12705T (I123I)	T14287C (G129G)<br>C14668T (M2M)		G8020A (P145P)<br>G8251A (G222G)	C8964T (T146T)	T14783C (L13L)<br>G15043A (G99G)<br>G15301A (L185L)
ColonLivM-3	–	-	Liver		C4883T (P138P)	C10400T (T114T)		T10873C (P38P)<br>G11719A (G320G)	C12705T (I123I)<br>T12957C (N207N)	C14668T (M2M)				T14783C (L13L)<br>G15043A (G99G)<br>A15301A (L185L)
ColonLivM-4	–	-	Liver		G5147A (T226T)<br>G5417A (Q316Q)		C10607T (L46L)	T11465C (L236L)<br>G11719A (G320G)	G12501A (M55M)<br>C12705T (I123I)<br>T13899C (Y562Y)					A14893G (L49L)
ColonLivM-5	–	-	Liver		T5108G (T29T)	C10400T (T114T)		T10873C (P38P)<br>G11719A (G320G)	C12705T (I123I)<br>A13563G (L409L)	T14200C (W158W)<br>G14569A (S35S)		G7600A (A5A)		T14783C (L13L)<br>G15043A (G99G)<br>G15301A (L185L)
ColonLivM-6	–	-	Liver		T4742C (N91N)			G11719A (G320G)				C7909T (Y108Y)		G15346A (L200L)<br>C15391T (S215S)
ColoLivM-8	67/M	Adenocarcinoma	Liver		A4769G (M100M)<br>A5084G (L205L)	C10400T (T114T)		G11719A (G320G)<br>G11914A (T385T)			Matched case 2		A9120G (L198L)	C15535T (N263N)
ColoCa-13LivM	66/M	Adenocarcinoma	Liver	G3970T (L222L)	A4769G (M100M)<br>C5049T (L194L)<br>G5147A (T226T)	G10310A (L84L)		C10976T (L73L)<br>G11719A (G320G)	C12633T (S99S)<br>C12882T (F182F)	C14476A (V66V)	Matched case 1	G7672A (M19M)<br>G8206A (M207M)		
ColonLuM-1	80/M	-	Lung			C10181T (F41F)<br>C10400T (T114T)		T10873C (P38P)<br>T11215T (Y152Y)		T14635C (V13V)<br>C14668T (M2M)		G8206A (M207M)		
ColonLuM-2<br>(LgCa203)	71/F	Adenocarcinoma	Lung			C10400T(T114T)		T10873C (P38P)<br>G11719A (G320G)		T14308C (G122G)		G8020A (P145P)		T14783C (L13L)<br>G15043A (G99G)<br>G15301A (L185L)<br>A15874G (M376M)

Supplementary Table S4. Properties of synonymous SNPs and SNVs in *ND* genes in NSCLC and colon cancer clinical samples.

Novel mutation

SNP/Mutation	Amino acid	Frequency T (this study) (%)	Frequency C (mtSNP-Japan)*1 (%)	Frequency (T/C)	No. of cases detected			
					NSCLC		Colon Cancer	
					Primary (N=45)	Metastases (N=37)	Primary (N=22)	Metastases (N=11)
**ND1**								
C3408T	R34R	1 /115 (0.87%)	0	-	0	0	1	0
G3483A	E59E	1/115 (0.87%)	0	-	0	1	0	0
C3495T	P63P	1/115 (0.87%)	0	-	0	0	1	0
G3531A	P75P	2/115 (1.74%)	3/672 (0.45%)	3.87	1	0	1	0
C3546T	T80T	1/115 (0.87%)	2/672 (0.30%)	2.9	1	0	0	0
T3552A	A82A	1/115 (0.87%)	1/672 (0.15%)	5.8	0	1	0	0
A3759G	L151L	1/115 (0.87%)	5/672 (0.74%)	1.18	1	0	0	0
T3826C	L174L	1/115 (0.87%)	1/672 (0.15%)	5.8	0	1	0	0
C3846T	P180P	1/115 (0.87%)	0	-	1	0	0	0
G3882A	E192E	2/115 (1.74%)	4/672 (0.60%)	2.9	0	1	1	0
C3921T	S205S	2/115 (1.74%)	0	-	2	0	0	0
C3970T	L222L	8/115 (6.96%)	40/672 (5.95%)	1.17	2	3	3 (1 w/ meta)	0
C4071T	Y255Y	7/115 (6.09%)	34/672 (5.06%)	1.2	3	2	2	0
T4117C	L271L	4/115 (3.48%)	15/672 (2.23%)	1.56	1	1	0	2
G4140T	P278P	2/115 (1.74%)	4/672 (0.60%)	2.9	1	1	0	0
A4164G	M286M	5/115 (4.35%)	30/672 (4.46%)	0.98	3	1	1	0
A4200T	L298L	1/115 (0.87%)	2/672 (0.30%)	2.9	1	0	0	0
T4248C	I314I	7/115 (6.09%)	50/672 (7.44%)	0.82	3	2	2 (1 w/ meta)	0
**ND2**								
C4505T	T12T	1/115 (0.87%)	9/672 (1.34%)	0.65	1	0	0	0
C4625T	S52S	2/115 (1.74%)	1/672 (0.15%)	11.6	1	1	0	0
G4655A	T62T	4/115 (3.48%)	39/672 (5.80%)	0.6	1	2	1 w/ meta	0
A4715G	G82G	2/115 (1.74%)	20/672 (2.98%)	0.34	1	1	0	0
T4742C	N91N	3/115 (2.61%)	4/672 (0.60%)	4.35	0	2	0	1
A4769G	M100M	115/115 (100%)	672/672 (100.00%)	1	45	37	22 (7 w/ meta)	11
A4793G	M108M	1/115 (0.87%)	22/672 (3.27%)	0.27	0	0	1	0
G4820A	E117E	6/115 (5.22%)	20/672 (2.98%)	1.75	2	1	2	1
C4850T	G127G	2/115 (1.74%)	4/672 (0.60%)	2.9	0	1	1	0
T4856C	L129L	1 /115 (0.87%)	0	-	0	1	0	0
T4859C	L130L	2/115 (1.74%)	12/672 (1.79%)	0.97	1	1	0	0
C4883T	P138P	35/115 (30.43%)	260/672 (38.69%)	0.79	17	9	7 (2 w/ meta)	2
A4895G	M142M	3/115 (2.61%)	13/672 (1.93%)	0.9	1	2	0	0
A4958G	M163M	11/115 (9.57%)	46/672 (6.85%)	1.4	5	4	1 w/ meta	1
G4991A	Q174Q	1/115 (0.87%)	1/672 (0.15%)	5.8	0	1	0	0
T5021C	I184I	2/115 (1.74%)	2/672 (0.30%)	5.8	1	0	1	0
C5049T	L194L	7/115 (6.09%)	15/672 (2.23%)	2.73	2	2	2 (1 w/ meta)	1
T5108C	T213T	3/115 (2.61%)	63/672 (9.38%)	0.28	1	0	1	1
G5147A	T226T	10/115 (8.70%)	41/672 (6.10%)	1.43	3	4	2 (1 w/ meta)	1
A5153G	L228L	4/115 (3.48%)	10/672 (1.49%)	2.34	2	1	1	0
G5231A	L254L	7/115 (6.09%)	38/672 (5.65%)	1.08	3	2	2	0
G5237A	P256P	2/115 (1.74%)	4/672 (0.60%)	2.92	1	0	1	0
G5261A	W264W	1/115 (0.87%)	4/672 (0.60%)	1.45	1	0	0	0
T5291C	N274N	1/115 (0.87%)	0	-	1	0	0	0
A5351G	L294L	5/115 (4.35%)	30/672 (4.46%)	0.98	3	1	1	0

G5417A	Q316Q	10/115 (8.70%)	55/672 (8.18%)	1.06	5	2	2	1
ND3								
C10104T	L16L	1/115 (0.87%)	14/672 (2.08%)	0.42	0	0	0	1
C10181T	F41F	1/115 (0.87%)	18/672 (2.68%)	0.32	0	0	0	1
T10235C	A59A	1/115 (0.87%)	2/672 (0.30%)	2.9	1	0	0	0
T10238C	I60I	1/115 (0.87%)	12/672 (1.79%)	0.49	1	0	0	0
G10310A	L84L	9/115 (7.83%)	50/672 (7.44%)	1.05	2	5	2 (1 w/ meta)	0
G10325A	M89M	1/115 (0.87%)	1/672 (0.15%)	5.8	0	0	1 w/ meta	0
A10397G	W113W	2/115 (1.74%)	29/672 (4.32%)	0.4	1	0	1	0
C10400T	T114T	54/115 (46.96%)	441/672 (65.62%)	0.72	21	18	8	7
A10403G	E115E	1/115 (0.87%)	1/672 (0.15%)	5.8	0	1	0	0
ND4L								
T10535C	Y22Y	1/115 (0.87%)	2/672 (0.30%)	2.9	0	0	1	0
G10586A	S39S	1/115 (0.87%)	2/672 (0.30%)	2.9	0	0	1	0
C10607T	L46L	2/115 (1.74%)	18/672 (2.68%)	0.65	1	0	0	1
G10646A	V59V	2/115 (1.74%)	6/672 (0.89%)	1.96	1	1	0	0
T10724C	Y85Y	1/115 (0.87%)	0	-	0	0	1	0
ND4								
T10790C	L11L	1/115 (0.87%)	4/672 (0.60%)	1.45	0	0	1	0
G10801A	L14L	2/115 (1.74%)	4/672 (0.60%)	2.9	2	0	0	0
T10873C	P38P	66/115 (57.39%)	440/672 (65.48%)	0.88	28	18	14 (5 w/ meta)	6
T10882C	F41F	1/115 (0.87%)	0	-	1	0	0	0
C10976T	L73L	6/115 (5.22%)	19/672 (2.83%)	1.84	2	1	2 (1 w/ meta)	1
A11017C	S86S	13/115 (11.30%)	38/672 (5.65%)	2	5	7	1	0
A11023G	P88P	1/115 (0.87%)	0	-	1	0	0	0
C11215T	Y152Y	9/115 (7.83%)	31/672 (4.61%)	1.7	6	0	2 (1 w/ meta)	1
A11239G	L160L	1/115 (0.87%)	0	-	1	0	0	0
T11260C	T167T	1/115 (0.87%)	0	-	1	0	0	0
C11389T	Y210Y	1/115 (0.87%)	1/672 (0.15%)	5.8	0	1	0	0
C11422T	V221V	1/115 (0.87%)	1/672 (0.15%)	5.8	0	0	1	0
T11437C	A226A	3/115 (2.61%)	5/672 (0.74%)	3.53	1	2	0	0
T11465C	L236L	1/115 (0.87%)	0	-	0	0	0	1
C11536T	Y259Y	7/115 (6.09%)	43/672 (6.40%)	0.95	4	2	1 w/ meta	0
T11569C	I270I	1/115 (0.87%)	1/672 (0.15%)	5.8	1	0	0	0
C11647T	L296L	6/115 (5.22%)	39/672 (5.80%)	0.9	3	2	1 w/ meta	0
C11665T	L302L	1/115 (0.87%)	4/672 (0.60%)	1.45	0	1	0	0
G11719A	G320G	111/115 (96.52%)	671/672 (99.85%)	0.97	44	36	22 (7 w/ meta)	9
T11722C	L321L	2/115 (1.74%)	6/672 (0.89%)	1.96	1	1	0	0
A11731G	S324S	1/115 (0.87%)	0	-	1	0	0	0
A11746G	L329L	1/115 (0.87%)	1/672 (0.15%)	5.8	0	0	1	0
G11914A	T385T	4/115 (3.48%)	37/672 (5.51%)	0.63	0	2	2 (1 w/ meta)	0
T11944C	L395L	2/115 (1.74%)	17/672 (2.53%)	0.69	0	1	1	0
G12007A	W416W	1/115 (0.87%)	4/672 (0.60%)	1.45	1	0	0	0
C12088T	P443P	2/115 (1.74%)	2/672 (0.30%)	5.8	1	1	0	0
T12091T	I444I	1/115 (0.87%)	4/672 (0.60%)	1.45	0	1	0	0
ND5								
A12358G	T8T	1/115 (0.87%)	0	-	1	0	0	0
G12372A	L12L	8/115 (6.96%)	38 672 (5.65%)	1.23	4	2	2	0
C12405T	L23L	5/115 (4.35%)	29/672 (4.32%)	1.01	3	1	1	0
G12501A	M55M	1/115 (0.87%)	10/672 (1.49%)	0.58	0	0	0	1
C12549T	T71T	2/115 (1.74%)	4/672 (0.60%)	2.9	1	1	0	0

C12633T	S99S	7/115 (6.09%)	18/672 (2.68%)	2.13	2	2	2 (1 w/ meta)	1
G12651C	L105L	3/115 (2.61%)	2/672 (0.30%)	8.7	1	2	0	0
C12705T	I123I	81/115 (70.43%)	545/672 (81.10%)	0.87	38	23	15 (4 w/ meta)	5
T12714C	I126I	1/115 (0.87%)	0	-	0	1	0	0
G12771A	E145E	11/115 (9.57%)	47 672 (6.99%)	1.37	5	4	2 (1 w/ meta)	0
C12853T	L173L	2/115 (1.74%)	1/672 (0.15%)	11.6	1	1	0	0
C12882T	F182F	9/115 (7.83%)	35/672 (5.21%)	1.5	2	3	2 (1 w/ meta)	2
T12957C	N207N	3/115 (2.61%)	4/672 (0.60%)	4.35	1	0	1	1
T13015C	L227L	1 /115 (0.87%)	0	-	0	1	0	0
C13029T	P231P	1/115 (0.87%)	1/672 (0.15%)	5.8	1	0	0	0
A13104G	G256G	3/115 (2.61%)	21/672 (3.12%)	0.84	0	2	1 w/ meta	0
A13152G	L272L	2/115 (1.74%)	4/672 (0.60%)	2.9	1	1	0	0
A13263G	Q309Q	1/115 (0.87%)	2/672 (0.30%)	2.9	0	1	0	0
A13278G	M315M	1/115 (0.87%)	8/672 (1.19%)	0.73	0	0	1	0
T13437C	P367P	3/115 (2.61%)	4/672 (0.60%)	4.35	2	1	0	0
A13563G	L409L	3/115 (2.61%)	32/672 (4.76%)	0.37	2	0	0	1
G13590A	L418L	4/115 (3.48%)	19/672 (2.83%)	1.23	2	1	0	1
T13602C	Y422Y	1/115 (0.87%)	2/672 (0.30%)	2.9	0	1	0	0
T13641C	P435P	3/115 (2.61%)	0	-	0	2	1 w/ meta	0
T13656C	L440L	2/115 (1.74%)	1/672 (0.15%)	11.6	2	0	0	0
G13707A	L457L	1/115 (0.87%)	0	-	0	0	1	0
C13782T	I482I	1/115 (0.87%)	0	-	0	0	1	0
A13887G	L517L	3/115 (2.61%)	4/672 (0.60%)	4.35	0	2	1 w/ meta	0
T13899C	Y562Y	1/115 (0.87%)	0	-	0	0	0	1
T13962C	L542L	2/115 (1.74%)	0	-	0	2	0	0
A14013G	E559E	1/115 (0.87%)	2/672 (0.30%)	2.9	0	0	1	0
C14067T	T577T	1/115 (0.87%)	0	-	0	0	1	0
A14133G	L599L	3/115 (2.61%)	12/672 (1.79%)	1.46	0	3	0	0
**ND6**								
T14200C	W158W	2/115 (1.74%)	30/672 (4.46%)	0.39	1	0	0	1
A14233G	D147D	1/115 (0.87%)	0	-	1	0	0	0
C14281T	G131G	1/115 (0.87%)	9/672 (1.34%)	0.65	1	0	0	0
T14287C	G129G	1/115 (0.87%)	10/672 (1.49%)	0.58	0	0	0	1
T14308C	G122G	4/115 (3.48%)	11/672 (1.64%)	2.12	1	2	0	1
T14319C	N119N	1/115 (0.87%)	0	-	1	0	0	0
G14364A	L104L	12/115 (10.43%)	42/672 (6.25%)	1.67	5	4	2 w/ meta	1
T14374C	V100V	1/115 (0.87%)	0	-	1	0	0	0
T14386C	L96L	1/115 (0.87%)	1/672 (0.15%)	5.8	1	0	0	0
A14464G	T70T	1/115 (0.87%)	0	-	1	0	0	0
T14470C	G68G	1/115 (0.87%)	29/672 (4.32%)	0.2	0	0	1 w/ meta	0
G14476A	V66V	7/115 (6.09%)	19/672 (2.83%)	2.15	2	2	3 (1 w/ meta)	0
G14569A	S35S	3/115 (2.61%)	64/672 (9.52%)	0.27	1	0	1	1
A14587G	G29G	1/115 (0.87%)	0	-	1	0	0	0
A14605G	P23P	2/115 (1.74%)	29/672 (4.32%)	0.23	0	2	0	0
T14635C	V13V	2/115 (1.74%)	0	-	1	0	0	1
C14668T	M2M	32/115 (27.83%)	237/672 (35.27%)	0.79	15	8	6 (2 w/ meta)	3

SNP/Mutation	Amino acid	Frequency T (this study) (%)	Frequency C (mtSNP-Japan)*1 (%)	Frequency (T/C)	No. of cases detected			
					NSCLC		Colon Ca	
					Primary (N=45)	Metastases (N=37)	Primary (N=22)	Metastases (N=11)
**COII**								
G7600A	A5A	2/115 (0.87%)	32/672 (4.76%)	0.18	1	0	0	1
G7672A	M19M	1/115 (0.87%)	0	-	0	0	0	1
T7684C	L33L	4/115 (3.48%)	29/672 (4.32%)	0.81	3	1	1	0
G7702A	L39L	1/115 (0.87%)	0	-	0	1	0	0
T7738C	T51T	1/115 (0.87%)	2/672 (0.30%)	2.9	0	1	0	0
T7741C	N52N	1/115 (0.87%)	0	-	0	1	0	0
C7747T	I55I	1/115 (0.87%)	0	-	0	0	1	0
T7783C	L67L	1/115 (0.87%)	0	-	0	0	1 w/ meta	0
A7828G	L81L	1/115 (0.87%)	2/672 (0.30%)	2.9	0	0	1	0
G7852A	E89E	1/115 (0.87%)	1/672 (0.15%)	5.8	0	1	0	0
C7867T	S94S	1/115 (0.87%)	25/672 (3.72%)	0.23	0	0	1	0
C7909T	Y108Y	2/115 (0.87%)	5/672 (0.74%)	1.18	0	1	0	1
C7948T	Y121Y	1/115 (0.87%)	2/672 (0.30%)	2.9	0	1	0	0
A7960G	P125P	1/115 (0.87%)	4/2704 (0.15%)	5.8	0	1	0	0
G8020A	P145P	6/115 (5.22%)	1/672 (0.15%)	34.8	1	2	1	2
T8167A	G194G	1/115 (0.87%)	84/672 (12.50%)	0.07	0	1	0	0
T8200C	S205S	1/115 (0.87%)	1/672 (0.14%)	6.2	0	0	1	0
G8206A	M207M	4/115 (3.48%)	25/672 (3.72%)	0.94	1	1	0	2
G8251A	G222G	1/115 (0.87%)	15/672 (2.23%)	0.39	0	0	0	1
**ATP6**								
T8598C	I24I	1/115 (0.87%)	0	-	1	0	0	0
T8614C	L30L	2/115 (0.87%)	1/672 (0.15%)	5.8	1	1	0	0
A8697G	M57M	1/115 (0.87%)	0	-	1	0	0	0
A8700G	M58M	1/115 (0.87%)	0	-	1	0	0	0
A8706G	M60M	1/115 (0.87%)	0	-	1	0	0	0
T8793C	P89P	2/115 (0.87%)	4/672 (0.60%)	1.45	1	1	0	0
C8829T	N101N	4/115 (3.48%)	25/672 (3.72%)	0.94	1	3	0	0
C8844T	I106I	1/115 (0.87%)	3/672 (0.45%)	1.93	0	1	0	0
G8856A	A110A	4/115 (3.48%)	5/672 (0.74%)	4.7	1	3	0	0
C8964T	T146T	5/115 (4.35%)	61/672 (9.08%)	0.48	1	2	1	1
A9120G	L198L	2/115 (0.87%)	1/672 (0.14%)	6.21	0	0	1 w/ meta	1
T9165C	V213V	1/115 (0.87%)	7/672 (1.04%)	0.84	1	0	0	0
A9180G	V218V	4/115 (3.48%)	27/672 (4.02%)	0.87	2	0	2	0
**CYTB**								
T14783C	L13L	65/115 (56.52%)	441/672 (65.62%)	0.86	29	18	13 (4 w/ meta)	5
A14893G	L49L	2/115 (0.87%)	18/672 (2.68%)	0.32	1	0	0	1
C14944T	I66I	2/115 (0.87%)	28/672 (4.17%)	0.21	2	0	0	0
T14968C	N74N	1/115 (0.87%)	21/672 (3.13%)	0.28	1	0	0	0
C15040T	I98I	1/115 (0.87%)	4/672 (0.60%)	1.45	1	0	0	0
A15043G	G99G	64/115 (55.65%)	442/672 (65.77%)	0.85	30	16	13 (3 w/ meta)	5
T15067C	F107F	6/115 (5.22%)	23/672 (3.42%)	1.53	3	2	1	0
G15106A	L120L	1/115 (0.87%)	3/672 (0.45%)	1.93	1	0	0	0
T15139C	Y131Y	2/115 (0.87%)	1/672 (0.15%)	5.8	2	0	0	0
G15148A	P134P	1/115 (0.87%)	6/672 (0.89%)	0.98	0	1	0	0
G15172A	G142G	1/115 (0.87%)	1/672 (0.15%)	5.8	1	0	0	0
T15184C	I146I	1/115 (0.87%)	6/672 (0.89%)	0.98	1	0	0	0

C15223T	D159D	4/115 (3.48%)	25/672 (3.72%)	0.94	1	3	0	0
A15301G	L185L	66/115 (57.39%)	441/672 (65.63%)	0.87	29	19	13 (4 w/ meta)	5
G15346A	L200L	7/115 (6.09%)	21/672 (3.13%)	1.95	1	5	0	1
C15391T	S215S	1/115 (0.87%)	3/672 (0.45%)	1.93	0	0	0	1
C15518T	L258L	1/115 (0.87%)	16/672 (2.38%)	0.37	0	0	1 w/ meta	0
C15535T	N263N	2/115 (0.87%)	21/672 (3.13%)	0.28	0	0	1 w/ meta	1
A15724G	W326W	1/115 (0.87%)	10/672 (1.49%)	0.58	0	0	1	0
A15874G	M376M	3/115 (2.61%)	23/672 (3.42%)	0.76	0	0	2 (1 w/ meta)	1

Somatic mutation. Novel mutations are shown in red.

*1 GiiB-JST mtSN; human mitochondrial genome single nucleotide polymorphyism database (http://mtsnp.tmig.or.jp/). SNP frequency in patients with Parkinson's disease, Altzheimer's disease, type 2 diabetes w/ and w/o vascular lesion, men w/ and w/o juvenile obese, and centenarians of Gifu and Tokyo.

Supplementary Table S5. Nonsynonemous SNPs and SNVs in the *ND* genes selected to evaluate the association with distant metastasis in NSCLC and colon cancer.

Gene	SNP/ Mutation*1	Amino acid substitution	Homoplasmy or heteroplasmy	Frequency (T/C)*2	Grantham value	Evolutionary conservation of the original amino acid	Conformational change	Reported disease association*3	MutPred Pathogenicity Score*4	No. of cases detected				No. of cases detected			
										NSCLC		Colon Cancer		Lung adenocarcinoma		Colon adenocarcinoma	
										Primary (n=45)	Metastases (n=37)	Primary (n=22)	Metastases (n=11)	Primary (n=45)	Metastases (n=37)	Primary (n=22)	Metastases (n=11)
ND1	T3394C	Y30H	Homoplasmy	2.09	83	Conserved		LHON / Diabetes / CPTdeficiency / high altitude adaptation	0.783	1	3	1 w/ distant meta	0	0	3	1 w/ distant meta	0
	T3398C	M31T	Homoplasmy	2.9	81	Conserved		DMDF+HCM / GDM / possibly LVNC cardiomyopathy-associated	0.751	0	0	1	0	0	0	0	0
	C3497T	A64V	Homoplasmy	2.04	64		Yes	LHON	0.413	1	5	0	1	1	5	0	0
	C3689G	A128G	Homoplasmy	-	60		Yes		0.747	0	1	0	1	0	1	0	1
	G3709A	A135T	Heteroplasmy or Heterogeneous	-	58	Conserved	Yes		0.774	1	0	0	0	0	0	0	0
	G3955A	A217T	Heteroplasmy or Heterogeneous	-	58		Yes		0.736	0	0	0	1	0	0	0	0
ND3	T10363C	L102P	Heteroplasmy or Heterogeneous	-	98	Conserved			0.746	0	1	0	0	0	1	0	0
ND4	C11409T	P217L	Heteroplasmy or Heterogeneous	-	98	Conserved			0.83	0	1	0	0	0	1	0	0
ND5	T12338C	M1T	Homoplasmy	3	81	Conserved		DEAF1555 increased penetrance / LHON	0.966	0	0	1	0	0	0	1	0
	C12813A	Y159STOP	Heteroplasmy or Heterogeneous	-	-		Truncated		-	0	1	0	0	0	1	0	0
	G13103A	G256E	Heteroplasmy or Heterogeneous	-	98	Conserved			0.834	1	0	0	0	0	0	0	0
	G13366A	G344STOP	Heteroplasmy or Heterogeneous	-	-		Truncated		-	0	0	1 (same case)	0	0	0	1 (same case)	0
	T14138C	L601P	Homoplasmy	-	98	Conserved			0.762	0	0	0	1	0	0	0	1
ND6	14504delA	L57STOP	Homoplasmy	-	-		Truncated		-	0	0	1 (same case)	0	0	0	1 (same case)	0

*1 Novel mutations found in this study are shown in red.

*2 Calculated on the basis of the SNP frequency (C) in persons with Parkinson's disease, Altzheimer's disease and type 2 diabetes w/ and w/o vascular lesion and men w/ and w/o juvenile obese, and centenarians of Gifu and Tokyo in the database of GiiB-JST mtSN; human mitochondrial genome single nucleotide polymorphyism database (http://mtsnp.tmig.or.jp/) and the SNP frequency (T) in this study.

*3 Data from MITOMAP database (http://www.mitomap.org/MITOMAP). LHON; Leber hereditary optic neuropathy, CPT; Carnitine Palmitoyltransferase, DMDF; Diabetes mellitus + Deafness, HCM; Hypertrophic cardiomyopathy, GDM; Gestational diabetes mellitus, LVNC; Left ventricular noncompaction.

*4 Predicted pathogenicity score (Ref. 18). The amino acid variants showing mor ethan 0.7 are considered as a high pathogenicity score group.

Supplementary Table S6. PCR primers used for sequencing and qPCR.

Primer	Sequence (5'-3')	Accession No.	Application
ND1-1F	AACAGGGTTTGTTAAGATGGC	NC_005089.1	Sequencing
ND1-1R	GGTTCGATTCTCATAGTCCTA		
ND1-2F	CATCAAACTCAAACTACGCCCT		
ND1-2R	TTCGGTTGGTCTCTGCTAGT		
ND2-1F	ATGAGAATCGAACCCATCCC		
ND2-1R	TAAGATTTTGCGTAGCTGGGT		
ND2-2F	GACATCCGGCCTGCTTCTT		
ND2-2R	GAGTGGGGTTTTGCAGTCC		
ND3-ND4L-F	CCATCTATTGATGAGGGTCTT		
ND3-ND4L-R	ATAATTAGGCTGTGGGTGGTT		
ND4-1F	CTAGTATATCGCTCACACCTCA		
ND4-1R	GCTTCGACATGGGCTTTAGGGA		
ND4-2F	CTACTCACTCTCACTGCCCAAG		
ND4-2R	GGGGAATTAGGGAAGTCAGGGT		
ND5-1F	CTCACAAGAACTGCTAACTCATGCC		
ND5-1R	CTAGTAGTGGGGTGAGGCTTGGATTA		
ND5-2F	CATCGGCTGAGAGGGCGTAGGAAT		
ND5-2R	GAGAGTAATAGATAGGGCTCAGGCG		
ND5-3F	CTCCGGGTCCATCATCCACAACCT		
ND5-3R	GATCCTATTGGTGCGGGGGCTTTG		
ND6-F	CAATTTCACAGCACCAAATCTCCA		
ND6-R	TATTAGGGGGTTAGTTTTGCG		
COII-F	ACACATTCGAAGAACCCGTAT		
COII-R	TTTAGTTGGGGCATTTCACTG		
ATP6-F	TAAATACTACCGTATGGCCCA		
ATP6-R	CGTTATGGAGTGGAAGTGAAA		
CYTB-F1	ACTAAACCCACACTCAACAGAAAG		
CYTB-F2	GTCATTAAGGAGAGAAGG		
CYTB-F2	ATACATTGGGACAGACCTAGTTC		
CYTB-R2	CTTTGGGTGCTAATGGTGGAGT		
HIF1a-F	CCATGCCCCAGATTCAAGATCA	NM_001313919.1	qPCR
HIF1a-R	TCACTGGGACTGTTAGGCTGG		
Pdh1a-F	CCTCCCTTTGAAGTGCGTGG	M76727.1	
Pdh1a-R	AGCACAGAGTTCCTTACGCT		
Pdhb-F	AAAGGCAAGGGACCCACATC	NM_024221	
Pdhb-R	TGGCTTCGATGTCCATTGGT		
Pdk1-F	AGGATCGGTTATGCCAACTGT	NM_172665	
Pdk1-R	AACCAGGAACTTCACACCCC		
MMP11-F	CACCGTCATCACCCTGTGAA	NM_008606.3	
MMP11-R	GGTTCCGGGCATTCAGTACA		
Plaur-F	ACTACCGTGCTTCGGGAATG	NM_011113.4	
Plaur-R	GCCTGTTGCAGAGGTTTGTG		
Ccl7-F	GATCTCTGCCACGCTTCTGT	NM_013654	
Ccl7-R	ATAGCCTCCTCGACCCACTT		
CD44-F	AGAAGAGCACCCCAGAAAGC	CT010324.1	
CD44-R	TCTGAAACCACGTCTCCTGC		
Kras-F	GCGCTGACCTAGGGAATGTT	NM_021284.6	
Kras-R	TCACTTCACAGCACGTACTCC		
Myc-F	ACAGTGTTCTCTGCCTCTGC	NM_001177352	
Myc-R	GCTGTACGGAGTCGTAGTCG		
Gapdh-F	TGCACCACCAACRGCTTAG	NM_008084	
Gapdh-R	GGATGCAGGGATGATGTTC		

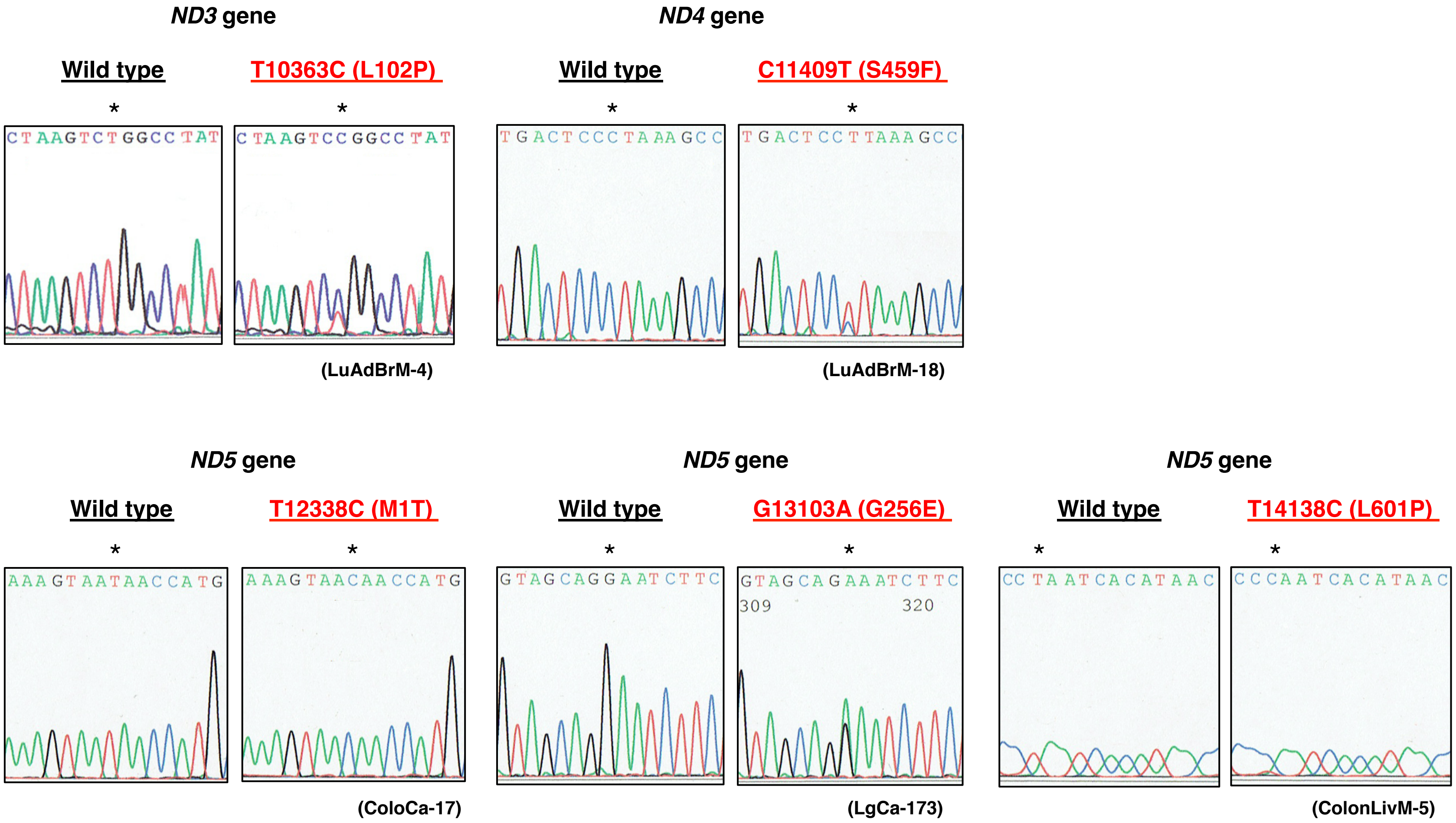

Supplementary Fig. S1. Selected SNVs found in the clinical samples.

T10363C

***ND3* a.a. sequences**	98 101	102	108 118
Homo sapiens	MSSLLLIIIL ALS	L	AYEWLQ KGLDWTE
**LuAdBrM-4 (L102P)**	MSSLLLIIIL ALS	P	AYEWLQ KGLDWTE
Mus musculus	IMAFILVTIL SLG	L	AYEWTQ KGLEWTE
Bos taurus	TMALFLIILL AVS	L	AYEWTQ KGLEWTE
Gallus gallus	TWATTIIALL TFG	L	IYEWTQ GGLEWAE
Xenopus laevis	LWAALILTLL TLG	L	IYEWLQ GGLEWAE
Salmo salar	AWSAAVLALL TLG	L	IYEWTG GGLEWAE
Drosophila melanogaster	ITSIIFILIL LIG	L	YHEWNQ GMLNWSN

C11409T

***ND4* a.a. sequences**	213 216	217	223 233
Homo sapiens	MVKMPLYGLH LWL	P	KAHVEA PIAGSMVLAA
**LuAdBrM-18 (P217L)**	MVKMPLYGLH LWL	L	KAHVEA PIAGSMVLAA
Mus musculus	LIKMPLYGVH LWL	P	KAHVEA PIAGSMILAA
Bos taurus	MVKMPLYGLH LWL	P	KAHVEA PIAGSMVLAA
Gallus gallus	MVKAPLYGLH LWL	P	KAHVEA PIAGSMLLAA
Xenopus laevis	MVKMPLYGTH LWL	P	KAHVEA PIAGSMVLAA
Salmo salar	LVKMPVYGVH LWL	P	KAHVEA PIAGSMILAA
Drosophila melanogaster	LVKMPMFLVH LWL	P	KAHVEA PVSGSMILAG

G13103A (G256E)

***ND5* a.a. sequences**	252 255	256	262 272
Homo sapiens	VSALLHSSTM VVA	G	IFLLIR FHPLAENSPL
**LgCa-173 (G256E)**	VSALLHSSTM VVA	E	IFLLIR FHPLAENSPL
Mus musculus	VSALLHSSTM VVA	G	IFLLVR FHPLTTNNNF
Bos taurus	VSALLHSSTM VVA	G	IFLLIR FYPLTENNKY
Gallus gallus	VSALLHSSTM VVA	G	IFLLIR THPFLSSNKT
Xenopus laevis	VSALLHSSTM VVA	G	IFLLIR ISPMMNNNQT
Salmo salar	VSALLHSSTM VVA	G	IFLLIR LHPLMENNQT
Drosophila melanogaster	VSALVHSSTL VTA	G	VYLLIR FNIILSTSWL

T14138C

***ND5* a.a. sequences**	590 600	601	
Homo sapiens	GMIKLYFLSP FFFPLVLTLL	L	IT
**ColoLivM-5 (L601P)**	GMIKLYFLSP FFFPLVLTLL	P	IT
Mus musculus	GLIKLYFMSN FLINIILIII	L	YSINLE
Bos taurus	GLIKLYFLST FLITILISMI	L	FNFHE
Gallus gallus	GLIKSYLGST FALTILTTIL	L	IQK
Xenopus laevis	GLIKTYLTLT FLMTSAIIIT	L	F
Salmo salar	GMIKTYLTLS FFLSTALAVL	L	TLT
Drosophila melanogaster	NSLKIYLLLW FVFWILILLI	L	LFL

Supplementary Fig. S2. Selected SNVs found in clinical samples that cause the substitution of evolutionarily highly conserved amino acids.
